# *Salmonella* Typhimurium discreet-invasion of the murine gut absorptive epithelium

**DOI:** 10.1371/journal.ppat.1008503

**Published:** 2020-05-04

**Authors:** Stefan A. Fattinger, Desirée Böck, Maria Letizia Di Martino, Sabrina Deuring, Pilar Samperio Ventayol, Viktor Ek, Markus Furter, Saskia Kreibich, Francesco Bosia, Anna A. Müller-Hauser, Bidong D. Nguyen, Manfred Rohde, Martin Pilhofer, Wolf-Dietrich Hardt, Mikael E. Sellin

**Affiliations:** 1 Institute of Microbiology, Department of Biology, ETH Zürich, Zürich, Switzerland; 2 Science for Life Laboratory, Department of Medical Biochemistry and Microbiology, Uppsala University, Uppsala, Sweden; 3 Institute of Molecular Biology & Biophysics, Department of Biology, ETH Zürich, Zürich, Switzerland; 4 Central Facility for Microscopy, Helmholtz Centre for Infection Research, Braunschweig, Germany; University of California, Davis, UNITED STATES

## Abstract

*Salmonella enterica* serovar Typhimurium (*S*.Tm) infections of cultured cell lines have given rise to the ruffle model for epithelial cell invasion. According to this model, the Type-Three-Secretion-System-1 (TTSS-1) effectors SopB, SopE and SopE2 drive an explosive actin nucleation cascade, resulting in large lamellipodia- and filopodia-containing ruffles and cooperative *S*.Tm uptake. However, cell line experiments poorly recapitulate many of the cell and tissue features encountered in the host’s gut mucosa. Here, we employed bacterial genetics and multiple imaging modalities to compare *S*.Tm invasion of cultured epithelial cell lines and the gut absorptive epithelium *in vivo* in mice. In contrast to the prevailing ruffle-model, we find that absorptive epithelial cell entry in the mouse gut occurs through “discreet-invasion”. This distinct entry mode requires the conserved TTSS-1 effector SipA, involves modest elongation of local microvilli in the absence of expansive ruffles, and does not favor cooperative invasion. Discreet-invasion preferentially targets apicolateral hot spots at cell–cell junctions and shows strong dependence on local cell neighborhood. This proof-of-principle evidence challenges the current model for how *S*.Tm can enter gut absorptive epithelial cells in their intact *in vivo* context.

## Author summary

Bacterial pathogens can use secreted effector molecules to drive entry into host cells. Studies of the intestinal pathogen *S*.Tm have been central to uncover the mechanistic basis for the entry process. More than two decades of research have resulted in a detailed model for how *S*.Tm invades gut epithelial cells through effector triggering of large Rho-GTPase-dependent actin ruffles. However, the evidence for this model comes predominantly from studies in cultured cell lines. These experimental systems lack many of the architectural and signaling features of the intact gut epithelium. Our study surprisingly reveals that in the intact mouse gut, *S*.Tm invades absorptive epithelial cells through a process that does not require the Rho-GTPase-activating effectors and can proceed in the absence of the prototypical ruffling response. Instead, *S*.Tm exploits another effector, SipA, to sneak in through discreet entry structures close to cell–cell junctions. Our results challenge the current model for *S*.Tm epithelial cell entry and emphasizes the need of taking a physiological host cell context into account when studying bacterium–host cell interactions.

## Introduction

Pathogenic Enterobacteriaceae, e.g. *Salmonella*, *Shigella*, and *Escherichia* species, cause >600 million cases of intestinal disease annually [1]. Many of these pathogens can be distinguished from the normal flora by their ability to invade the intestinal epithelium. Bacterial invasion elicits a mucosal inflammatory response, diarrheal symptoms, and in some cases systemic bacterial spread [2].

Studies of the broad spectrum pathogen *Salmonella enterica* serovar Typhimurium (*S*.Tm) have been instrumental for our understanding of bacterial host cell invasion strategies [3,4]. Current knowledge derives largely from *S*.Tm infections of mammalian cell lines (e.g. HeLa, MDCK, Caco-2, Cos7) grown on cell culture plastic. Under such conditions, *S*.Tm uses flagella-driven near-surface swimming to reach the edges of target cells [5]. Subsequently, the bacterium deploys a needle-like Type-Three-Secretion System (TTSS-1) encoded by *Salmonella* Pathogenicity Island-1 (SPI-1), which, assisted by a set of adhesins, promotes docking to the host cell [3]. Insertion of the SipBC translocon–present at the TTSS-1 tip–into the host cell membrane initiates translocation of TTSS-1 effectors into the cytosol [6]. Three effectors, namely SopB, SopE, and SopE2, have been shown to constitute the main drivers of epithelial cell line invasion [7–12]. SopB carries a lipid phosphatase activity that alters the phosphatidylinositol-phosphate (PIP) composition of the plasma membrane inner leaflet [13]. This indirectly recruits multiple host factors, including Rho-GTPases and their activating Guanine nucleotide Exchange Factors (GEFs) to sites of bacterial docking [14,15]. SopE and SopE2 mimic cellular GEFs to directly activate actin-regulatory GTPases, such as Rac1 and Cdc42 [8,9]. In combination, SopBEE2 activate a cohort of Rho- and Arf family GTPases, resulting in WAVE-regulatory complex (WRC) and Arp2/3-dependent actin nucleation, and the induction of large lamellipodia- and filopodia-containing membrane ruffles for bacterial uptake [8,9,14,16,17] (SopBEE2 hereafter referred to as “ruffle-inducers”). The ruffles also fuel macropinocytic uptake of bystander bacteria–a phenomenon referred to as cooperative invasion [5,18,19].

Acquisition of SPI-1 has been an early event in the evolution of pathogenic *Salmonellae* [20]. Notably, SopB, SopE, and SopE2 are all encoded on genetic elements outside of SPI-1. Hence, genes encoding the ruffle-inducers have likely been acquired by separate horizontal gene transfer events and the proteins incorporated into the TTSS-1 arsenal. SPI-1 itself, however, also harbors primordial TTSS-1 effector genes. The translocon component SipC can promote actin polymerization, at least *in vitro* [21,22]. Moreover, the SipA effector gene is located within SPI-1 and can be found across *Salmonella* serovars [23]. The SipA protein can directly bind to, stabilize, and protect bundled actin, but does not induce large ruffles on its own [22,24–26]. Intriguingly, deletion of SipA results in only a subtle impact on invasiveness and entry structure morphology in cell line culture experiments [25,27,28]. Consequently, the contribution of the conserved SipA effector to epithelial cell invasion has been regarded as minor [4].

While the molecular details of *S*.Tm invasion into cultured cell lines have been resolved to a considerable detail, studies of the invasion process under physiological infection conditions remain rare. In the intestine of permissive mice, gut absorptive epithelial cells constitute prominent targets for *S*.Tm invasion [29–31]. By sharp contrast to tumor-derived cell lines, the absorptive intestinal epithelium *in vivo* exhibits i) the signaling properties of primary non-transformed cells, ii) a strictly polarized columnar cell arrangement, iii) dense apical microvilli, iv) a high degree of cellular interconnectedness, v) a heterogeneous set of neighboring epithelial cell types, and vi) a luminal barrier comprised of antimicrobial peptides, mucus, and commensal microbes [32]. As cell line infection assays were used to establish the prevailing model for *S*.Tm epithelium invasion, the implications of most of these physiological features remain ill-defined.

Experiments in *in vivo* models of salmonellosis have addressed the impact of bacterial virulence factors and host defenses on mucosal tissue pathology. In the frequently used streptomycin-pretreated mouse model [33,34], per-oral infection studies have revealed a contribution of the TTSS-1 apparatus, the TTSS-1 effectors SopE, SopE2, and SipA as well as the SiiE adhesin encoded on SPI-4 to *S*.Tm-inflicted mucosal pathology at ~1–3 days post-infection (p.i.) [35–38]. Multiple TTSS-1 effectors, including in addition SopA, SopB, and SopD, contribute to diarrheal symptoms also during bovine infection [39]. Some morphological work has also been conducted to probe the epithelial invasion step *in vivo*, in mucosal tissue explants, or in ligated loops from e.g. guinea pig, calf, mouse or pig [40–44]. These studies have highlighted *S*.Tm invasion of both microfold cells (M cells) and absorptive epithelial cells in Peyer’s patch regions of the mucosa, and identified a variety of morphological host cell features connected to *S*.Tm entry [41–44]. Additionally, recent work in neonate mice has revealed that the effectors SipA and SopE2 work redundantly to promote *S*.Tm invasion of, and traversal through, immature gut epithelial cells during the first days of the infection [45]. Nevertheless, comprehensive studies of how *S*.Tm invades the mature gut epithelium of adult hosts during the first critical hours of acute infection remain scarce.

Our earlier work in the streptomycin mouse model established that *S*.Tm targets cecal absorptive epithelial cells in substantial numbers during the initiating phase of acute infection [29,30]. Here, as well as in the small intestine of infected neonate mice, *S*.Tm rapidly forms intraepithelial microcolonies [30,31]. These microcolonies could largely be explained by replication, i.e. not by entry of several bacteria into the same host cell [30,31]. Since co-invasion of multiple bacteria is a hallmark of ruffle-mediated entry in cultured cell lines [5,18,19], these observations hinted that the model for *S*.Tm epithelial cell invasion might not be transferable to the intact gut epithelium.

Here, we have undertaken a comparative analysis of *S*.Tm invasion into common epithelial cell lines and the murine gut absorptive epithelium *in vivo*. Our work reveals that *S*.Tm invades gut absorptive epithelial cells through “discreet-invasion”, a mode that differs markedly from ruffle-invasion of epithelial cell lines. Discreet-invasion critically requires TTSS-1 translocation of the primordial effector SipA, induces modest lengthening of local microvilli in the absence of expansive ruffles, does not support cooperative *S*.Tm entry, targets the apicolateral region of infected epithelial cells in a neighbor-dependent manner, and results in swift normalization of the epithelial cell brush border. These findings challenge the accepted model for how *S*.Tm enters gut epithelial cells and prompt further *in vivo* studies across the diversity of *Salmonella* strains and host species.

## Results

### *S*.Tm invasion of epithelial cell lines and the murine gut absorptive epithelium differentially requires SPI-4 and the TTSS-1 effectors SopB, SopE, SopE2, and SipA

**Fig 1:**
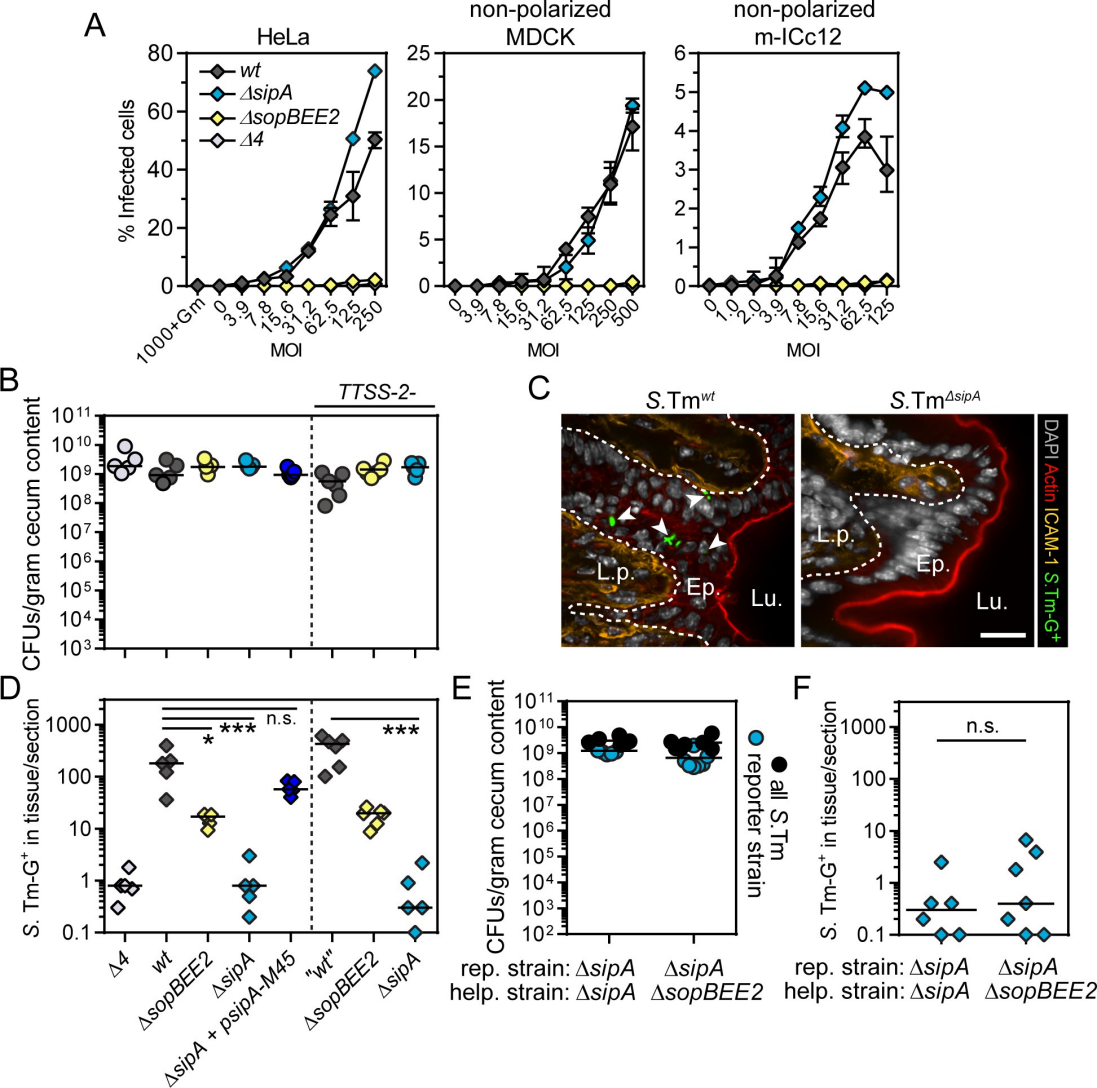
*S*.Tm invasion of cultured epithelial cell lines and the murine gut absorptive epithelium differentially depends on bacterial virulence factors. (**A**) SopBEE2 drive *S*.Tm invasion of non-polarized epithelial cell lines. Invasion efficiency of *S*.Tm/p*ssaG-GFP* reporter strains in the indicated subconfluent epithelial cell lines, infected for 20min over a range of MOIs, and analyzed at 4h p.i. by automated microscopy. Data points correspond to mean +/- range of two to three replicate infections. (**B-D**) SipA drives *S*.Tm gut absorptive epithelial cell invasion *in vivo* in mice. Sm-pretreated C57BL/6 wild-type mice were orally infected with the indicated *S*.Tm/p*ssaG-GFP* reporter strains for 12h. The three strains to the right of the dashed line are also deficient for TTSS-2. (**B**) *S*.Tm CFU counts in cecum content. (**C**) Representative micrographs of the cecal mucosa upon infection with the indicated strains. Lu.—Lumen; Ep.—Epithelium; L.p.—Lamina propria. White arrow heads indicate intraepithelial *S*.Tm. Scale bar: 10μm. (**D**) Quantification of intraepithelial *S*.Tm per 20μm section. In B and D each data point corresponds to one animal. Line at median. Kruskal-Wallis with Dunn’s post-test (n.s., not significant; *p<0.05; ***p<0.001). (**E-F**) SipA cannot promote epithelial cell invasion when present on a co-infecting bacterium. Wild-type mice were infected with a 1:1 mix of the indicated *S*.Tm/p*ssaG-GFP*-reporter strain (rep.) and non-fluorescent helper strain (help.) for 12h. (**E**) *S*.Tm CFU counts in cecum content. (**F**) Quantification of intraepithelial *S*.Tm per 20μm section. Each data point in E-F corresponds to one animal. Line at median. Mann-Whitney U-test (n.s., not significant).

Efficient *S*.Tm invasion of epithelial cell lines requires TTSS-1 function [46]. In addition, invasion of polarized MDCK cells involves the SiiA-E adhesin system encoded on SPI-4, which is dispensable for entry into non-polarized cell lines (S1A–S1C Fig) [47]. In *S*.Tm^*wt*^ (SL1344), the TTSS-1 effectors SopB, SopE, and SopE2 have been shown to predominantly drive ruffle-mediated entry, while the actin-binding effector SipA has a less prominent role [8,9,17,27]. We used automated microscopy to explore the generality of these findings in non-polarized epithelial cell lines of human (HeLa), canine (sub-confluent MDCK), and murine origin (m-ICc12). Joint deletion of the ruffle-inducers (i.e. *ΔsopBEE2*) essentially abolished *S*.Tm invasive behavior in all cases, similar to deletion of all four effectors (i.e. deletion of *sopBEE2sipA*;“*Δ4”*) (Fig 1A). By contrast, we were unable to detect a significant invasion defect upon deletion of SipA (*ΔsipA*) in either cell line (Fig 1A). In fact, at high pathogen densities, *S*.Tm^*ΔsipA*^ invaded HeLa and m-ICc12 marginally better than *S*.Tm^*wt*^. These data, supported by bacterial plating assays (S1D Fig), validate the ruffle-inducers SopBEE2 as the key drivers of *S*.Tm invasion into diverse non-polarized epithelial cell lines.

Due to its well-defined colonization kinetics, we chose the streptomycin mouse model of *Salmonella* gut infection to study *S*.Tm invasion of the intestinal epithelium *in vivo*. In this model, luminal colonization and epithelial cell invasion begins in the cecum [34]. We investigated if the first wave of invasion shows a similar timing throughout this entire gut segment. C57BL/6 wild-type mice were infected (by oral gavage; 5x10^7^ CFU) with *S*.Tm^wt^ harboring a p*ssaG-GFP* reporter (turns GFP+ subsequent to host cell entry [36]). Microscopy scoring of tissue-lodged *S*.Tm-GFP+ revealed equivalent numbers of intracellular bacteria across the entire cecal length (S1E and S1F Fig). As expected, the vast majority of *S*.Tm invasion foci (~95%) localized to EpCam-positive absorptive epithelial cells (S1G and S1H Fig). Hence, by microscopy of the middle part of the cecum, we can sensitively quantify *S*.Tm invasion of gut absorptive epithelial cells *in vivo*. As a baseline, we infected wild-type mice for 8-12h with *S*.Tm^wt^ or isogenic strains deficient in TTSS-1 (*ΔinvG*) or the Sii adhesin system (*Δspi-4*), all harboring the p*ssaG-GFP* reporter. *S*.Tm^*wt*^ were as expected abundant in the cecal epithelium, whereas the *S*.Tm^*ΔinvG*^ strain exhibited virtually undetectable numbers of invasion events (~200-fold attenuated at 8h; >1000-fold attenuated at 12h p.i.; S1J–S1L Fig). Deletion of *spi-4* resulted in ~20-fold lower levels of tissue-residing bacteria at 8h, but only a minor difference at 12h p.i. (S1J–S1L Fig). Consequently, early invasion of the naïve murine gut absorptive epithelium critically relies on TTSS-1 and is further enhanced by the SPI-4-encoded adhesin system.

Next, we infected wild-type mice (orally as above) with *S*.Tm^*wt*^, *S*.Tm^*ΔsipA*^, *S*.Tm^*ΔsopBEE2*^ and *S*.*Tm*^*Δ4*^ strains to analyze the dependence on TTSS-1 effectors. All strains equally colonized the gut lumen at 12h p.i. (Fig 1B). Remarkably, and in sharp contrast to results from the cell lines (Fig 1A), the *S*.Tm^*ΔsipA*^ strain reached dramatically lower pathogen densities in the gut epithelium than *S*.Tm^*wt*^ (~200-fold lower; Fig 1C and 1D). *S*.Tm^*ΔsopBEE2*^ was also attenuated, but still ~10-20-fold more invasive than *S*.Tm^*ΔsipA*^ (Fig 1D). In fact, the epithelial loads of *S*.Tm^*ΔsipA*^ were similar to those of the strain lacking all four effectors (*S*.Tm^*Δ4*^; Fig 1D), or the TTSS-1 apparatus itself (*S*.Tm^*ΔinvG*^; S1L Fig). Plasmid complementation restored SipA protein expression and epithelial invasion (Fig 1D and S1M Fig). Finally, similar observations were also made in a strain background lacking a functional TTSS-2 apparatus, which is important for intracellular *S*.Tm life (Fig 1D, right panels). Taken together, our data reveal a fundamentally different impact of TTSS-1 effectors on *S*.Tm invasion into epithelial cell lines—primarily driven by the ruffle-inducers SopBEE2 (Fig 1A), vs the murine gut absorptive epithelium *in vivo*—primarily driven by SipA (Fig 1B–1D).

SipA also affects *S*.Tm-induced gut inflammation (S1N Fig). Based on prior proposals [48], it remained conceivable that SipA could indirectly facilitate bacterial access to the epithelium *in vivo*, through inflammation-induced thwarting of protective barriers (i.e. affecting immune cell influx, mucus structure, or antimicrobial peptide production). If that was the case, then co-infection with a separate SipA-expressing strain should help *S*.Tm^*ΔsipA*^ achieve wild-type invasion efficiency. However, while a co-administered SipA-proficient helper strain (*S*.Tm^*ΔsopBEE2*^) augmented the early signs of inflammation (median pathoscore increased from 1 to 5; S1O Fig), it did not increase the invasion efficiency of a *S*.Tm^*ΔsipA*^ reporter strain (Fig 1E and 1F; compare with SipA plasmid complementation in 1D). We conclude that SipA needs to be expressed by the invading bacterium itself to drive epithelial cell entry *in vivo*.

One feature that distinguishes the gut absorptive epithelium from common cell lines is its confluent, columnar, polarized cell arrangement. Epithelial cell polarization may be influenced by and/or affect the impact of *S*.Tm effectors [49,50]. We investigated if this property could account for the exquisite dependence on SipA for *S*.Tm invasion of the gut epithelium. MDCK cells were cultured in parallel as subconfluent non-polarized vs confluent polarized cell layers on cell culture plastic (S2A Fig). *S*.Tm^*ΔsipA*^ showed equal invasion efficiency as *S*.Tm^*wt*^ into non-polarized MDCK cells, but a ~2-3-fold attenuation in polarized MDCK cells (S2B and S2C Fig). To assess if this observation could be generalized, we cultured mouse m-ICc12 cells and human Caco-2 C2Bbe1 cells atop Transwell inserts. In agreement with previous work [47,51], m-ICc12 cells only formed a semi-polarized/tight monolayer (judged by trans-epithelial electrical resistance; TEER<50 Ωcm2) (S2D Fig), while Caco-2 C2Bbe1 cells formed a tightly polarized monolayer (TEER>1000Ωcm2; S2E Fig). SipA was dispensable for *S*.Tm invasion also of semi-polarized m-ICc12 cells (S2F Fig). During short-term infection of polarized Caco-2 C2Bbe1 cells, the invasion capacity of *S*.Tm^*ΔsipA*^ was however reduced ~2-fold compared to *S*.Tm^*wt*^ (S2G Fig). Hence, SipA may moderately boost *S*.Tm invasion of polarized epithelial cells in culture (S2 Fig), but this feature cannot by itself account for the ~200-fold impact of the SipA effector on epithelial cell invasion in the mouse gut (Fig 1C and 1D).

In summary, *S*.Tm invasion of the murine gut absorptive epithelium is facilitated by the SPI-4 adhesin system and critically depends on TTSS-1 delivery of the primordial effector SipA, with a surprisingly small contribution of the three ruffle-inducers SopBEE2.

### Experiments in inflammasome-deficient mice verify the importance of SipA for gut absorptive epithelium invasion

**Fig 2:**
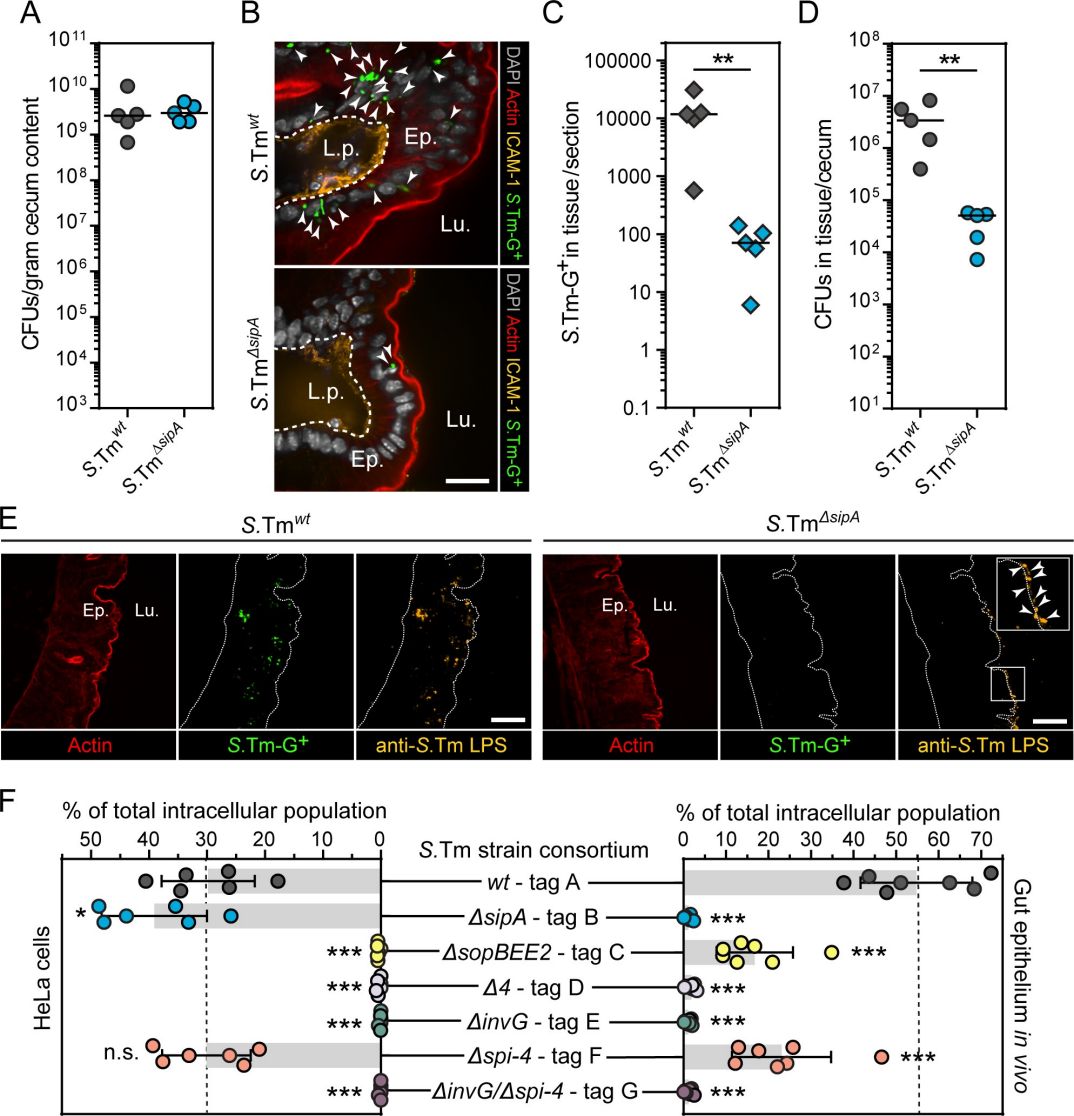
Infections in inflammasome-deficient mice confirm the dependence on SipA for *S*.Tm absorptive epithelial cell invasion *in vivo*. (**A-E**) Deletion of SipA has a profound impact on absorptive epithelial cell invasion in the mouse gut. Inflammasome-deficient (*Nlrc4*^*-/-*^) mice were orally infected with the indicated *S*.Tm/p*ssaG-GFP* reporter strains for 18h. (**A**) *S*.Tm CFU counts in cecum content. (**B**) Representative micrographs of the cecal mucosa upon infection with the indicated strains. Lu.—Lumen; Ep.—Epithelium; L.p.—Lamina propria. White arrow heads indicate intraepithelial *S*.Tm. Scale bar: 10μm. (**C**) Microscopy-based quantification of intraepithelial *S*.Tm per 20μm section. (**D**) *S*.Tm CFU counts in cecal mucosa tissue. In A, C, and D each data point corresponds to one animal. Line at median. (C-D) Mann-Whitney U-test (**p<0.01). (**E**) Representative micrographs of the extensively washed cecal mucosa. Blow-up in rightmost panel highlights *S*.Tm^*ΔsipA*^ on the epithelial surface (white arrow heads). Lu.—Lumen; Ep.—Epithelium. Scale bar: 50μm. (**F**) Barcoded consortium infections reveal the differential dependence on bacterial virulence factors for *S*.Tm invasion of epithelial cell lines vs. the gut absorptive epithelium in mice. Relative abundance of the indicated barcoded *S*.Tm strains in the intracellular population in HeLa cells (left panel; 20min infection at MOI 0.2–2) and *in vivo* in the cecal mucosa of *Nlrc4*^*-/-*^ mice (right panel; infected as in A above). Bars correspond to mean +/- SD of six replicate infections pooled from two different occasions (left panel) or infection of seven replicate mice (right panel). Each replicate is indicated by a circle symbols. One-way ANOVA with Dunnett´s test (n.s., not significant; *p<0.05, ***p<0.001).

Several TTSS-1 effectors, and in particular SipA, have been shown to shape the intracellular *S*.Tm niche and/or promote the bacterium’s replicative potential subsequent to host cell entry [45,52–54]. This could potentially skew the results above that rely on the p*ssaG-GFP* reporter to quantify invasion efficiency *in vivo*. Moreover, infected epithelial cells in wild-type mice are frequently and quickly expelled into the lumen by an inflammasome response [30,55,56]. This reduces pathogen loads in the mucosal tissue and might hamper precise quantification of epithelial cell invasion efficiencies. To substantiate the observations above, we conducted *S*.Tm infections in inflammasome-deficient (*Nlrc4*^*-/-*^) mice [57]. This allowed infections for a longer time period (18h) without overt epithelium destruction and resulted in ~50-100-fold higher total bacterial numbers in the cecal absorptive epithelium (Fig 2A–2D and S1I Fig). At these high loads, robust tissue plating experiments could be performed without problems arising from carry-over contamination by the dense luminal *S*.Tm population (approximately 10^9^ cfu/g content; Figs 1B and 2A).

We first infected *Nlrc4*^*-/-*^ mice for 18h with *S*.Tm^*wt*^ or *S*.Tm^*ΔsipA*^ harboring the p*ssaG-GFP* reporter. The strains colonized the gut lumen equally (Fig 2A), but *S*.Tm^*ΔsipA*^ again exhibited ~100-fold lower total numbers of epithelium-lodged GFP+ bacteria (Fig 2B and 2C). Enumerating the number of infected cells per section instead of the total *S*.Tm load yielded similar results, i.e. 100-200-fold lower numbers of epithelial cells infected by *S*.Tm^*ΔsipA*^ (S3A Fig). Moreover, virtually identical results were also obtained upon gentamycin treatment of the mucosal tissue followed by plating of total intracellular bacteria (Fig 2D). Finally, staining of permeabilized cecal sections with anti-*Salmonella*-LPS antibodies revealed plenty of tissue-residing *S*.Tm^*wt*^ (Fig 2E), whereas *S*.Tm^*ΔsipA*^ invasion foci were virtually absent from the epithelial tissue (Fig 2E). Instead, *S*.Tm^*ΔsipA*^ were found enriched on the epithelial surface (Fig 2E, rightmost panel and insert). These results exclude that the effect of SipA on epithelial *S*.Tm loads *in vivo* can be explained by altered reporter maturation, or by a SipA effect on replication (although we do not refute that such effects could also exist). Of further note, intraepithelial *S*.Tm foci on average contain only a low number (mean ~2) of bacteria during the first 12-18h of infection ([30]; see also Fig 5E below). This means that the frequency of invasion events, rather than intraepithelial replication, predominantly governs the intraepithelial *S*.Tm load during early gut infection. Taken together, our results support that SipA deletion results in normal *S*.Tm gut lumen colonization, normal approach of and binding to the gut epithelium, but a profound epithelial cell invasion defect.

*In vivo* infections are subject to large animal-to-animal variations. Furthermore, in infections with one individual mutant per mouse, mutants attenuated at an early step of the infection process may face delayed onset of host defense and thereby grow or survive differently than *S*.Tm^*wt*^. This could complicate scoring of attenuation phenotypes *in vivo*. To substantiate the contribution of *S*.Tm effectors to epithelial cell invasion under internally controlled conditions, we employed our recently developed method for barcoded consortium infections [19]. Unique inert 40-nucleotide tags (informed by [58]) were placed on the bacterial chromosome of each strain of interest and infections conducted with a mixed inoculum comprising equal amounts of each strain. Quantitative PCR of genomic DNA was employed to quantify the relative abundance of each tag in enrichment cultures of the input (inoculum or luminal content) and output (intracellular) bacterial populations (see materials and methods for details).

For the consortium infections, we employed seven isogenic barcoded strains: *S*.Tm^*wt*^-tag A, *S*.Tm^*ΔsipA*^-tag B, *S*.Tm^*ΔsopBEE2*^-tag C, *S*.Tm^*Δ4*^-tag D, *S*.Tm^*ΔinvG*^-tag E, and the additional control strains *S*.Tm^*Δspi-4*^-tag F, and *S*.Tm^*ΔinvGΔspi-4*^-tag G (S1 Table). These strains were mixed in a 1:1:1:1:1:1:1 ratio and used as inoculum for infections in both cultured epithelial cell lines and the *in vivo* model. Quantitative PCR showed similar abundances of each strain in the inoculum (S3B Fig). However, in the population successfully invading HeLa cells (MOI 0.2–2; 20min infection), the relative strain abundance was shifted significantly. *S*.Tm^*ΔsipA*^-tag B and *S*.Tm^*Δspi-4*^-tag F were present at roughly equal levels than *S*.Tm^*wt*^-tag A (Fig 2F, left panel). By contrast, *S*.Tm^*ΔsopBEE2*^-tag C, *S*.Tm^*Δ4*^-tag D, *S*.Tm^*ΔinvG*^-tag E, and *S*.Tm^*ΔinvGΔspi-4*^-tag G were all close to undetectable (<1% of total bacterial population). Similar results were also obtained in infections of m-CIc12 cells on plastic and of polarized Caco-2 C2Bbe1 cells grown atop Transwell inserts (S3C and S3D Fig). Using a less complex consortium, we at shorter infection times (7-10min) detected ~1.5-2-fold attenuated invasion of *S*.Tm^*ΔsipA*^ in polarized Caco-2 C2Bbe1 (S3E Fig). These results are in agreement with results from automated microscopy and bacterial plating assays above (Fig 1A, S1A Fig, S1D Fig and S2F and S2G Fig).

Next, we infected *Nlrc4*^*-/-*^ mice with the same seven-strain consortium and extracted the luminal and epithelial tissue-residing bacterial populations. This yielded the expected total bacterial loads in both compartments (~10^9^ *S*.Tm/g cecum content, S3F Fig; and ~10^6^ *S*.Tm in tissue/cecum, S3G Fig). *S*.Tm^*wt*^-tag A outperformed the other strains with respect to epithelial invasion (~55% of the intracellular population; Fig 2F right panel). *S*.Tm^*Δspi-4*^-tag F exhibited a modest attenuation (~23% of intracellular population) compared to the wild-type. Importantly, deletion of SipA (*S*.Tm^*ΔsipA*^*—*tag B) again resulted in a dramatic loss in invasiveness (≤1% of total intracellular population), whereas the strain lacking the ruffle-inducers (*S*.Tm^*ΔsopBEE2*^—tag C) performed markedly better (~16% of total intracellular population) (Fig 2F right panel). Taken together, multiple experimental approaches demonstrate that *S*.Tm invasion of the murine gut absorptive epithelium critically depends on TTSS-1 translocation of the primordial effector SipA. This contrasts starkly to observations of the invasion process in cultured epithelial cell lines.

### *S*.Tm invades gut absorptive epithelial cells through discreet entry structures

The results above (Figs 1 and 2) indicate that SipA is a key driver of epithelial cell invasion *in vivo*, in the absence or presence of SopBEE2. Importantly, previous work has suggested that SipA on its own is incapable of inducing large ruffles in cultured cell lines (e.g. [26,59]). The strong SipA-dependence for gut epithelium entry raises the question whether the model for *S*.Tm invasion through large SopBEE2-dependent ruffles applies to the *in vivo* scenario. We hypothesized that the primary, differentiated, polarized, and neighbor-connected nature of absorptive epithelial cells *in vivo* steer *S*.Tm invasion towards a SipA-dependent, and away from a ruffle-dependent, entry mechanism. We therefore examined the presence and morphology of entry structures around invading *S*.Tm in cell lines exhibiting different degrees of polarization, and in the mouse gut.

**Fig 3:**
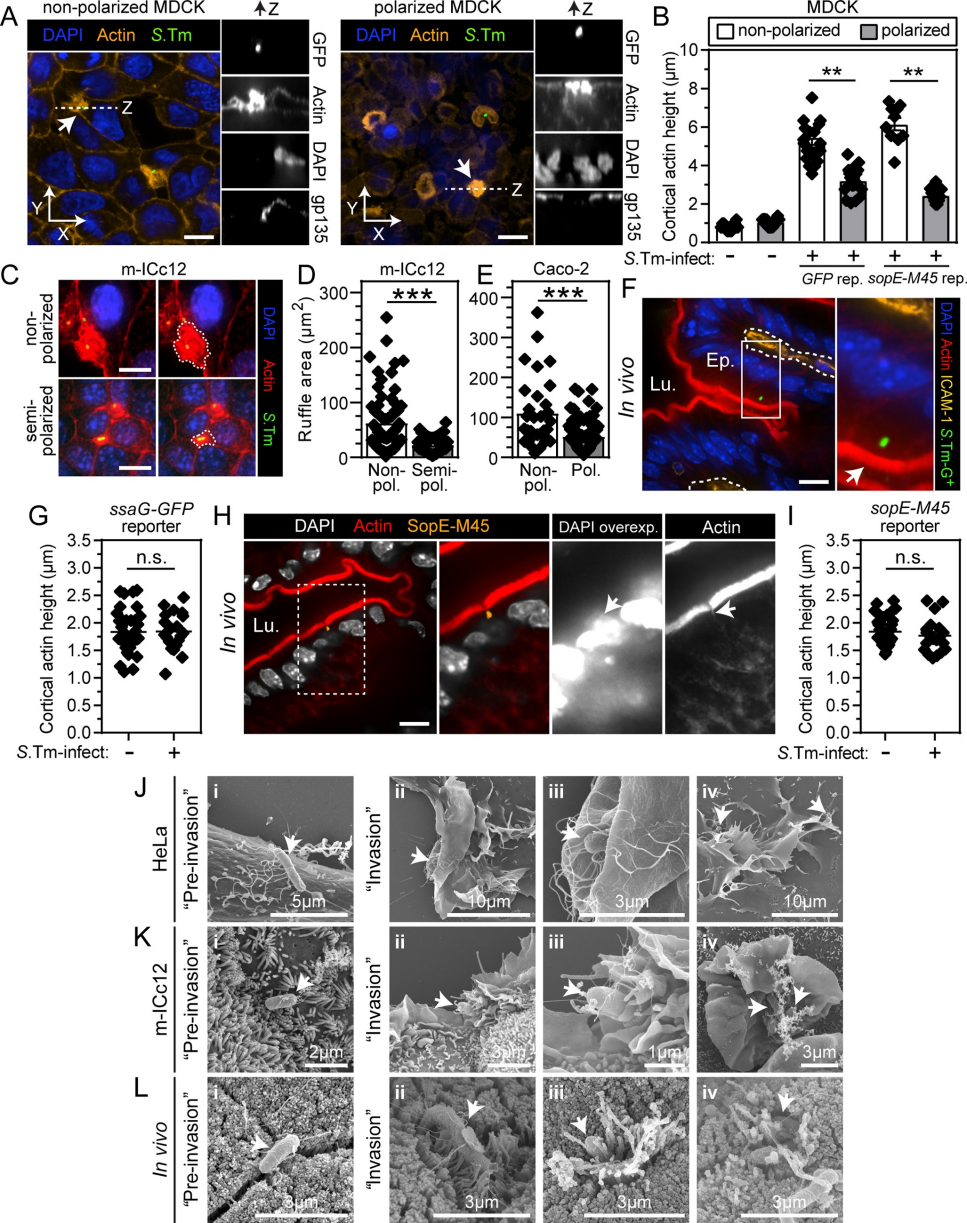
*S*.Tm-induced entry structures differ between epithelial cell lines and the absorptive gut epithelium in mice. (**A-E**) Epithelial cell polarization status affects the size of *S*.Tm-induced actin ruffles. (**A**) Confocal microscopy Z-stack visualizations of non-polarized and polarized MDCK cells infected with *S*.Tm^*wt*^/p*GFP*. Scale bar: 10μm. (**B**) Quantification of cortical actin height at invasion foci in MDCK cells infected with *S*.Tm^*wt*^/p*GFP* or *S*.Tm^*wt*^/p*sopE-M45*. (**C**) Representative maximum intensity projection micrographs of non-polarized and semi-polarized m-ICc12 cells grown atop Transwell inserts and infected with *S*.Tm^*wt*^/p*mCherry* (green pseudocoloring). White dashed line indicate *S*.Tm-induced entry structures. Scale bars: 10μm. (**D-E**) Quantification of *S*.Tm-induced ruffle area in (D) non-polarized and semi-polarized m-ICc12 cells and (E) non-polarized and polarized Caco-2 C2Bbe1 cells grown atop Transwell inserts. (**F-I**) Intact actin brush border without signs of actin ruffle formation in the *S*.Tm-infected gut absorptive epithelium *in vivo*. (**F-G**) Wild-type mice were orally infected with *S*.Tm^*wt*^ /p*ssaG-GFP* for 6h. (**F**) Representative micrographs of the cecal epithelium. Blow-ups show magnifications of boxed regions. Lu.—Lumen; Ep.—Epithelium. White arrows indicate the apical actin brush border of an infected cell. Scale bar: 10μm. (**G**) Quantification of actin brush border height in infected cells and uninfected neighbors. (**H-I**) *Rag1*^*-/-*^ mice (used since detection of M45 relies on a mouse monoclonal) were orally infected with *S*.Tm^*wt*^/p*sopE-M45* for 8h. (**H**) Representative micrographs of a SopE-M45 positive focus in the cecal epithelium. Blow-up shows magnification of boxed region. Lu.—Lumen. White arrow indicates M45-positive bacterial focus. Scale bar: 10μm. (**I**) Quantification of actin brush border height in infected cells and uninfected neighbors. Each data point in B, D, E, G, and I corresponds to one (non-infected or infected) cell. Bar or line represents mean. Results are representative for two to three experiments in each case. Mann-Whitney U-test (n.s., not significant; **p<0.01; ***p<0.001). (**J-L**) Different ultrastructural appearance of *S*.Tm entry structures in epithelial cell lines and the gut absorptive epithelium in mice. (**J-K**) Representative SEM micrographs of (J) HeLa cells and (K) m-ICc12 cells infected with *S*.Tm^*wt*^ for 6-10min at MOI 400. (**L**) Representative SEM micrographs of the cecal epithelium of mice infected with *S*.Tm^*wt*^. Arrows indicate *S*.Tm. Scale bars as indicated in each panel.

We began by characterizing *S*.Tm entry structures in MDCK cells, cultured in parallel either as flat-growing or polarized cell layers on plastic. The cells were infected with *S*.Tm^*wt*^ expressing constitutive GFP (pM965; S1 Table) and infections terminated by fixation. We consistently noted induction of ~3.5–8μm high actin ruffles in non-polarized MDCK cells (Fig 3A and 3B). Polarized MDCK cells produced significantly smaller entry structures (~2–4.5 μm height) in response to *S*.Tm^*wt*^/p*GFP* (Fig 3A and 3B). In agreement with previous work [27], these ruffles were also shaped differently, often with a circular appearance when viewed from the top. Similar results were obtained with an *S*.Tm strain expressing a SopE-M45 reporter protein (S1 Table), which allowed focusing the analysis on host cells that recently experienced delivery of TTSS-1 effectors (Fig 3B). Moreover, when m-ICc12 and Caco-2 C2Bbe1 cells were grown as semi-polarized/polarized monolayers atop Transwell inserts (see growth conditions in S2D and S2E Fig), they produced smaller ruffles upon *S*.Tm infection, as compared to their subconfluent non-polarized counterparts (Fig 3C–3E). These data show that polarization of epithelial cells reduces their propensity to generate large ruffles in response to *S*.Tm docking and effector translocation.

High loads of luminal bacteria complicate the use of constitutive reporters to visualize epithelial cell invasion *in vivo*. Using *S*.Tm/p*ssaG-GFP* in mice, we did not observe overt perturbation of the apical actin brush border of infected epithelial cells at the resolution of light-microscopy (Fig 3F and 3G, S4A Fig). This could imply that i) no ruffles are forming, or that ii) *in vivo* entry structures are exquisitely short-lived and the apical actin returns to normal before the *ssaG-GFP*-reporter produces visible fluorescence. To resolve this uncertainty, we again utilized the *S*.Tm^*wt*^/p*sopE-M45* reporter strain. Of note, translocated effector proteins can by this approach be detected within less than a minute of TTSS-1 secretion [60]. Indeed, SopE-M45 could be detected as a deposit in occasional infected epithelial cells *in vivo* (Fig 3H and S4B Fig). Since this effector exhibits a short half-life within host cells (half-life << 1h; [61]), the approach allowed us to focus the analysis of entry structures to epithelial cells that had recently experienced TTSS-1 effector translocation. Notably, we again did not detect any pronounced perturbations of the actin brush border of infected cells compared to uninfected neighbors (Fig 3H and 3I, S4B Fig).

Next, we used scanning electron microscopy (SEM) to investigate the ultrastructural appearance of *S*.Tm entry sites. As expected, *S*.Tm inoculums contained rod-shaped ~0.5–1μm wide and ~2μm long bacteria with peritrichous flagella (S5A Fig). In HeLa cells, large membrane ruffles accompanied 48% of all cell-associated *S*.Tm^*wt*^ (N = 128) (Fig 3J panel ii-iv, S5B Fig). The remainder of the events did not involve direct contact between the bacterial body and the host cell, and thus likely represent pre-invasion stages (Fig 3J, panel i). *S*.Tm-induced ruffles were typically ~10–20μm in diameter and featured large lamellipodial structures (Fig 3J, panels ii-iii, S5B Fig), combined with filopodial protrusions (Fig 3J, panel iv; S5B Fig). Infected mouse m-ICc12 cells produced somewhat smaller ruffles, but still containing both lamellipodial and filopodial elements and reaching diameters of up to ~10μm (Fig 3K).

A parallel SEM analysis of the intact murine gut epithelium (of *S*.Tm^*wt*^-infected mice) provided no evidence of expansive membrane ruffles. Instead, *S*.Tm^*wt*^-associated gut epithelial cells featured either no signs of cell surface perturbation (Fig 3L, panel i; S5C Fig, top right panels), or only discreet ultrastructural effects (Fig 3L, panels ii-iv; S5C Fig, bottom panels). Where host cell binding was evident, *S*.Tm engaged in multivalent interactions with, and distortion of, proximally located microvilli (Fig 3L, panel ii; S5C Fig, bottom panels). In some further progressed invasion events, a rim of elongated, bent, and deformed microvilli emerged around the bacterium (Fig 3L, panel iii; S5C Fig, bottom panels). Finally, *S*.Tm captured in late stages of invasion displayed a “sinking-in” appearance surrounded by a mix of normal and distorted microvilli (Fig 3L, panel iv; S5C Fig, bottom panels). Importantly, no large lamellipodial ruffles were observed (scrutiny of the cecal mucosa in N = 5 mice). From these data, we conclude that *S*.Tm enters the intact gut absorptive epithelium through discreet entry structures, distinct from the expansive SopBEE2-dependent ruffles observed in epithelial cell lines. This agrees with an entry mechanism dominated by TTSS-1 delivery of the primordial effector SipA (Figs 1 and 2). We term this mode of *S*.Tm entry into the murine gut epithelium “discreet-invasion”.

### SipA drives ruffle-independent invasion into a tight vacuolar compartment

We next aimed to resolve the dynamic features of SipA-driven epithelial cell invasion at high temporal resolution. We began by infecting non-polarized epithelial cell lines, i.e. sub-confluent MDCK and HeLa cells expressing LifeAct (for actin visualization), with fluorescent *S*.Tm strains. At the end of the time-series, fixation and anti-*S*.Tm-LPS staining without prior permeabilization allowed us to determine which pathogen-host cell encounters had led to successful invasion.

**Fig 4:**
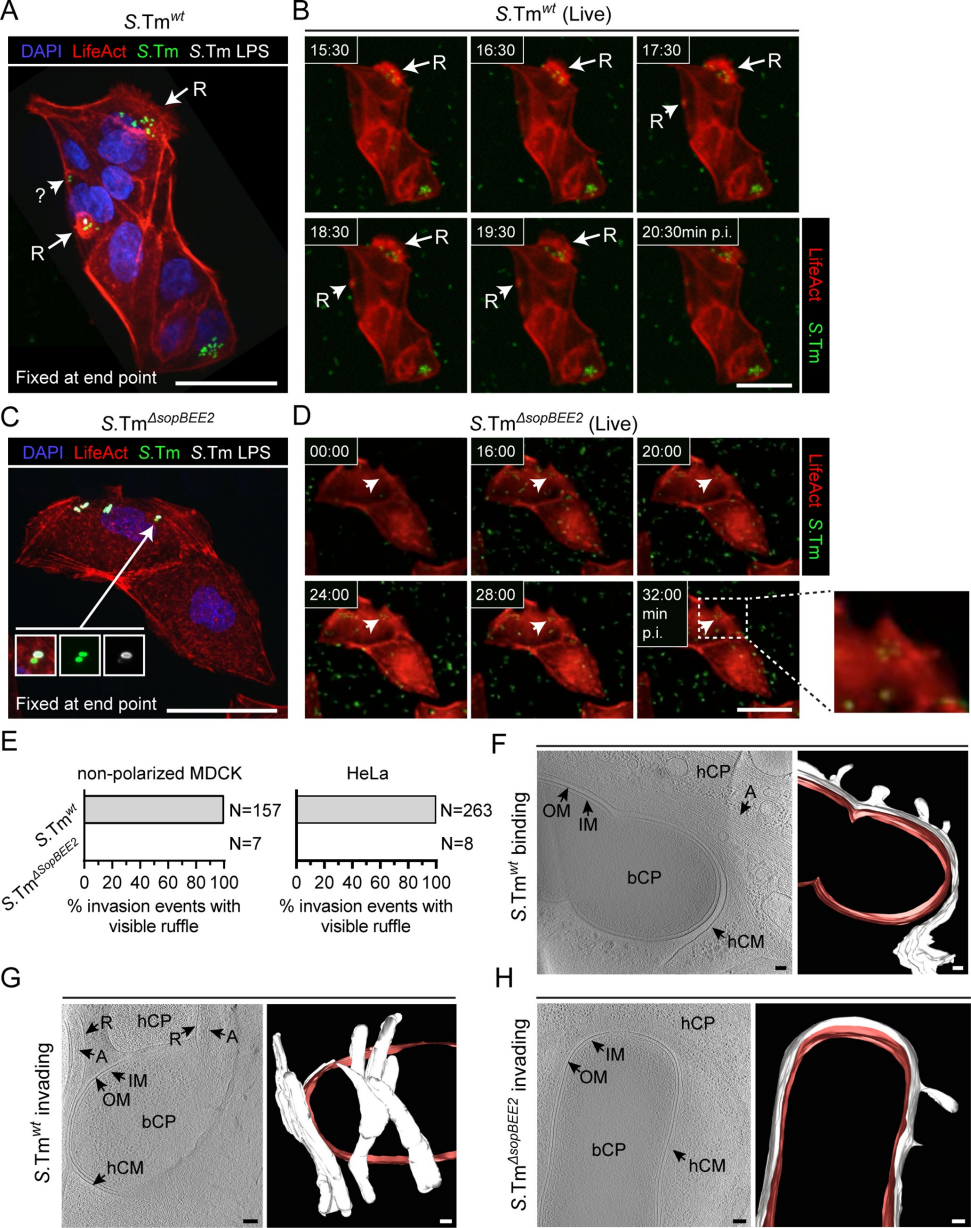
SipA drives *S*.Tm epithelial cell invasion without triggering higher order actin meshworks. (**A-E**) By contrast to *S*.Tm^*wt*^, *S*.Tm^*ΔsopBEE2*^ invades non-polarized epithelial cells in the absence of visible ruffles. (**A-B**) Non-polarized LifeAct-expressing MDCK cells (red) were infected with *S*.Tm^*wt*^/p*mCherry* (green) at MOI 50. (**A**) Representative micrograph of cells fixed at the end point (24min p.i.). Successful invasion was scored by α-*S*.Tm LPS-staining prior to permeabilization. R–ruffle ;? —ambiguous entry event revealed as a ruffle in the live series. (**B**) Live imaging series preceding A. Arrow/Arrow heads labelled R indicate extensive and transient ruffles, respectively. (**C-D**) MDCK cells as in A-B were infected with *S*.Tm^*ΔsopBEE2*^/p*mCherry* (green) at MOI 500. (**C**) Representative micrograph of cells fixed and stained at the end point (40min p.i.). (**D**) Live imaging series preceding C. Arrow head indicates a ruffle-less entry focus. Scale bars in A-D: 20μm. (**E**) Live imaging-based quantification of the presence/absence of ruffles at *S*.Tm^*wt*^ and *S*.Tm^*ΔsopBEE2*^ entry sites in non-polarized MDCK (left; N_tot_ = 164 invasion events) and HeLa (right graph; N_tot_ = 271 invasion events analyzed). (**F-H**) Ultrastructural underpinnings of SipA-driven epithelial cell invasion. Cryo-electron tomograms of HeLa cells infected with *S*.Tm^*wt*^ (F-G; N_tot tomograms_ = 20, frozen at 14min p.i.), or *S*.Tm^*ΔsopBEE2*^ (H; N_tot tomograms_ = 13; frozen at 40min p.i.). Shown in each case is a 15-17nm slice of a tomogram and the respective segmentation. R—ruffle; OM—outer membrane; IM—inner membrane; bCP—bacterial cytoplasm; hCP—host cell cytoplasm; hCM—host cell membrane; A—actin filaments; white pseudo-coloring—host cell membrane; red pseudo-coloring—bacterial membrane. Scale bars in F-H: 100 nm.

Initial experiments showed that *S*.Tm^*wt*^ always entered non-polarized MDCK cells (N = 157/157) and HeLa cells (N = 263/263) through clearly visible actin ruffles ((Fig 4A, 4B and 4E and S1 Movie (non-polarized MDCK) and S2 Movie (HeLa)). Ruffle duration (from 4min to persistence over the whole 24min series) and size (~5–15μm diameter in MDCK cells) varied greatly, however. The strain invading through SipA (i.e. *S*.Tm^*ΔsopBEE2*^) has a poor capacity to enter cultured cell lines (Fig 1A–1C), which prompted us to carry out the corresponding live experiments at a high MOI over a longer time period (MOI 500, 40min). Nevertheless, all *S*.Tm^*ΔsopBEE2*^ invasion events detected in both cell types occurred in the complete absence of actin ruffles (Fig 4C–4E and S3 Movie).

To survey the impact of epithelial cell polarization, we visualized a large number of *S*.Tm^*wt*^ invasion events (N = 524) in real-time in polarized LifeAct-expressing MDCK cells. Microscopy of samples fixed at the end point of infection highlighted abundant intracellular *S*.Tm (S6A Fig). Analysis of the preceding live-imaging series revealed that a majority of invasion events (94,8%) were surrounded by the pronounced actin signal enrichment expected from a ruffling response (S6A–S6C Fig; indicated by arrows; S4 Movie). Notably, however, a fraction (5,2%) of invasion events displayed no signs of actin ruffles around the invading bacterium at any time of the movie (S6A–S6C Fig; encircled in white; S4 Movie). These data indicate that wild-type *S*.Tm can in fact enter polarized epithelial cells without triggering marked actin-dependent ruffles. Analogous experiments showed that the *S*.Tm^*ΔsopBEE2*^ strain always entered polarized MDCK cells without the emergence of actin ruffles (N = 24/24) (S6D–S6F Fig; indicated by arrow heads; S5 Movie). Hence, SipA drives ruffle-independent epithelial cell invasion regardless of epithelial host cell polarity or context (Fig 4C–4E, S6D–S6F Fig).

Cryo-electron tomography of the thin edges of plunge-frozen HeLa cells was used to determine the ultrastructural underpinnings of the wild-type and “SipA-only”-driven invasion processes in a near-native state. The cryo-tomograms illustrated abundant actin bundles underneath *S*.Tm^*wt*^ captured at early stages of host cell binding (S6 Movie and Fig 4F). For events captured at a later stage of invasion, these bundles were replaced by a multidirectional meshwork of actin filament-rich protrusions around the bacterium (S7 Movie and Fig 4G; red pseudo-coloring delineates the bacterial membrane and white pseudo-coloring the host cell membrane). Again by sharp contrast, invading *S*.Tm^*ΔsopBEE2*^ bacteria were found within a tightly wrapped and smooth membrane compartment (S8 Movie and Fig 4H). Hence, the *S*.Tm^*ΔsopBEE2*^ strain, which invades specifically through SipA, enters non-polarized epithelial host cells by sinking into a tight vacuole without inducing complex higher-order actin meshworks. This is especially notable since non-polarized epithelial cells are highly permissive for ruffling if exposed to bacteria expressing SopB, SopE and/or SopE2. Taken together, our time-lapse and 3D-reconstruction data provide a direct link between the primordial TTSS-1 effector SipA and a discreet-invasion mode for epithelial cell entry.

### Non-cooperative *S*.Tm invasion into the murine gut absorptive epithelium

Cooperative invasion, i.e. that an actively invading bacterium promotes the entry also of bystander bacteria, is prevalent in cultured cell lines infected with *S*.Tm [5,18,19]. Expansive membrane ruffles elicited by the primary invader generates physical obstacles where secondary motile bacteria get entangled and taken up [5]. Cooperative invasion depends on the likelihood of secondary bacteria finding a ruffle and will therefore increase in frequency with higher MOI, larger ruffle size, and/or longer ruffle duration [5,18,19]. As such, cooperative invasion provides a functional readout for *S*.Tm-elicited host cell ruffling responses.

**Fig 5:**
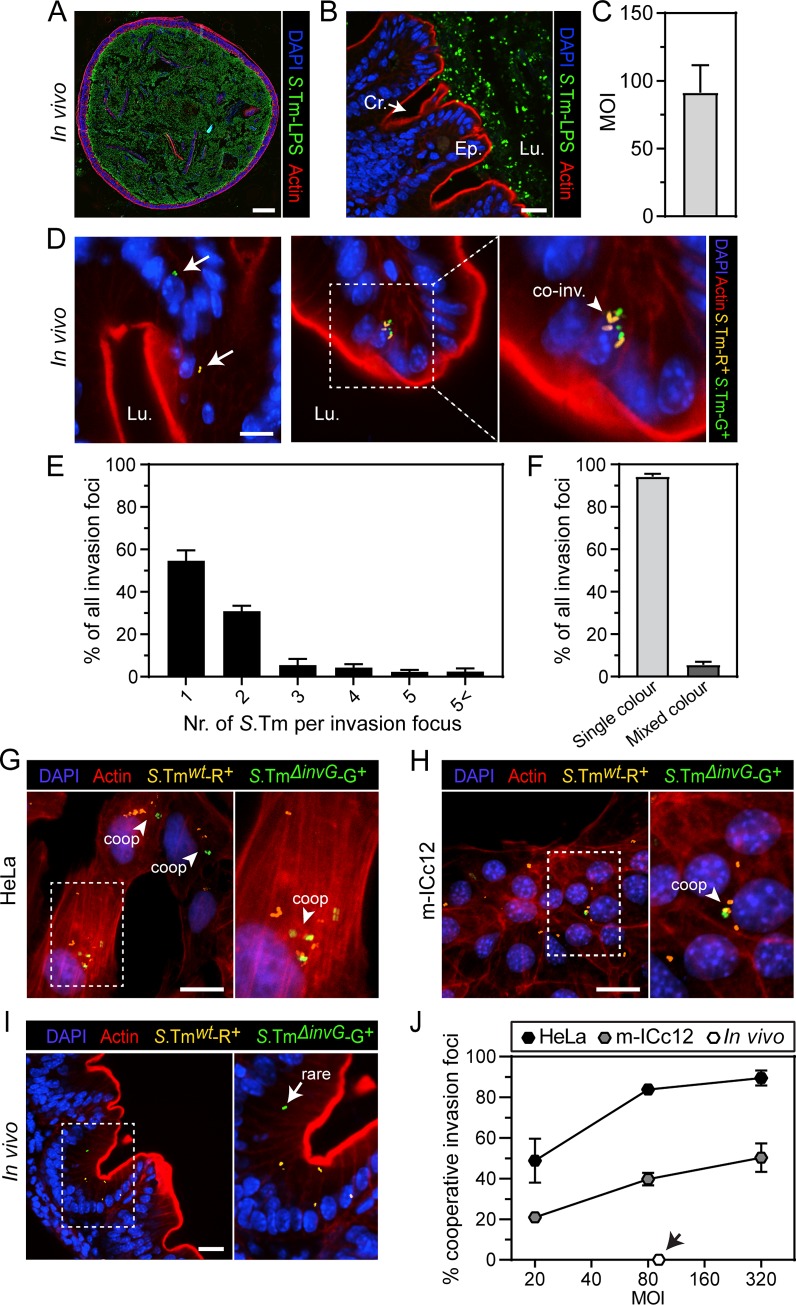
Cooperative *S*.Tm invasion occurs frequently in epithelial cell lines, but not in the absorptive gut epithelium of mice. (**A-C**) *S*.Tm Multiplicity of Infection (MOI) in the gut of (Sm-pretreated) orally infected mice. Inflammasome-deficient (*Nlrc4*^*-/-*^) mice were orally infected with *S*.Tm^*wt*^ for 12h. (**A**) Representative stitched image of an entire cross-section of the infected murine cecum. Scale bar: 500μm. (**B**) Representative up-close micrograph of the infected cecum lumen and mucosa. Lu.—Lumen; Ep.—Epithelium; Cr.—Crypt. Scale bar: 20μm. (**C**) Microscopy-based quantification of the MOI in the infected mouse cecum. Bar correspond to mean +/- SD of replicate infections in four mice. (**D-F**) *S*.Tm co-invasion occurs rarely in the mouse gut. Inflammasome-deficient (*Nlrc4*^*-/-*^) mice were orally infected with a 1:1 mix of *S*.Tm^*wt*^/p*ssaG-mCherry* and *S*.Tm^*wt*^/p*ssaG-GFP* strains for 12h. (**D**) Representative micrographs of the cecal epithelium. Blow-up shows magnification of boxed region. Lu.—Lumen. White arrows indicate one-coloured invasion foci; arrow head labelled “co-inv” indicates a mixed invasion focus. Scale bar: 10μm. (**E**) Quantification of the distribution of bacterial numbers within *S*.Tm invasion foci. (**F**) Quantification of the frequency of single-colour and mixed colour invasion foci *in vivo*. Bars in E-F correspond to mean +/- SD of replicate infections in four mice (N_tot_ = 1055 invasion events analyzed). (**G-J**) Prevalent cooperative *S*.Tm invasion in epithelial cell lines, but not in the absorptive gut epithelium *in vivo*. (**G-H**) Infection of (G) HeLa and (H) m-ICc12 cells with a 1:1 mix of *S*.Tm^*wt*^/p*ssaG-mCherry* and *S*.Tm^*ΔinvG*^/p*ssaG-GFP* strains for 1h at MOI 80. Representative micrographs. Blow-ups show magnifications of boxed regions. Arrow heads labelled “coop” indicate cooperative invasion foci. Scale bar: 20μm. (**I**) Inflammasome-deficient (*Nlrc4*^*-/-*^) mice were orally infected with a 1:1 mix of *S*.Tm^*wt*^/p*ssaG-mCherry* and *S*.Tm^*ΔinvG*^/p*ssaG-GFP* strains for 12h. Representative micrographs of the cecal epithelium. Blow-up shows magnification of boxed region. Arrow points to a rare *S*.Tm^*ΔinvG*^/p*ssaG-GFP* invasion focus. Scale bar: 20μm. (**J**) Quantification of the frequency of cooperative invasion foci in HeLa, m-ICc12 cells (infection parameters as in G-H) and the absorptive gut epithelium *in vivo* (infection parameters as in I). Data points correspond to mean +/- SD of three replicate infections in HeLa and m-ICc12 cells, and to mean +/- SD of five mice (N_tot_ = 578 *in vivo* invasion events analyzed), respectively.

In the mouse cecum, the mucus barrier covers the crypts, whereas the top ~50% of the epithelial layer is in contact with motile luminal *S*.Tm [62]. Based on this prior knowledge, we used confocal microscopy to estimate the effective MOI in the cecum at 12h p.i. (in *Nlrc4*^*-/-*^ mice to prevent epithelium distortion). We quantified the total luminal *S*.Tm population and the number of accessible epithelial cells per section. *S*.Tm were evenly spaced over the gut lumen cross-section with a modest enrichment at the epithelial border (Fig 5A). As expected, only few bacteria localized within crypts, while *S*.Tm were frequently found in contact with the differentiated part of the gut epithelium (Fig 5B). Repeated analysis resulted in an MOI estimation of 91+/-20 (mean+/-SD; Fig 5C). It should here be noted that luminal *S*.Tm loads in wild-type and *Nlrc4*-deleted mice are equal during early infection (Figs 1B and 2A) [30]. Moreover, the gut luminal pathogen population reaches a stable plateau of colonization (~10^8^−10^9^ CFUs/gram content) already at 6-8h p.i. (S1J Fig) [30]. Hence, the naïve mouse cecal epithelium experiences close contact with a dense and motile luminal *S*.Tm population for several hours during acute infection.

We infected *Nlrc4*^*-/-*^ mice with a 1:1 mix of two differentially labelled wild-type *S*.Tm strains (*S*.Tm^*wt*^/p*ssaG-mCherry* and *S*.Tm^*wt*^/p*ssaG-GFP*) to begin assessing cooperative invasion frequency. At 12h p.i. ~55% of all epithelial invasion foci carried only one bacterium, ~30% carried two bacteria, and the remaining foci carried three bacteria or more (Fig 5D and 5E). Most notably, only ~5% of all invasion foci carried a mix of green and red bacteria (Fig 5D–5F; N_tot_ = 1055 foci in 4 mice analyzed). This subfraction of invasion foci could either have resulted from co-invasion (i.e. two active invasion events into the same host cell, occurring in parallel or in sequence), or alternatively from true cooperative (helped) invasion. In either case, the results show that at an estimated MOI of 91+/-20 in the mouse gut, cooperative epithelial cell invasion is at best rare.

To estimate the frequency of true cooperative invasion events, we adapted the dual-colored mixed inoculum to include one actively invading strain (*S*.Tm^*wt*^/p*ssaG-mCherry*) and one strain incapable of TTSS-1-mediated active entry (*S*.Tm^*ΔinvG*^/p*ssaG-GFP*; >1000-fold reduced invasion capacity in single strain infections; S1L Fig). A mixed invasion focus could with this setup only arise if the *S*.Tm^*wt*^ strain promoted cooperative entry of *S*.Tm^*ΔinvG*^. As reference, the mixed inoculum was used to infect cultured HeLa and m-ICc12 cells at MOIs spanning across the range noted *in vivo* (MOI 20–320, 1h infection). HeLa cells produce expansive *S*.Tm-induced ruffles (Fig 3J), and as expected cooperative invasion was highly prevalent at all MOIs tested (Fig 5G–5J). At an MOI close to the *in vivo* estimate (MOI 80) >80% of all invaded cells carried *S*.Tm of both colors (Fig 5J). Mouse m-ICc12 cells produce somewhat smaller ruffles (Fig 3K), and consequently cooperative invasion was less common than in HeLa cells, but still comprised ~40% of all invasion events at MOI 80 (Fig 5H–5J). By stark contrast, we did not observe a single case of cooperative epithelial cell invasion in the mouse cecum (Fig 5I and 5J; N_tot_ = 578 foci in 5 mice analyzed). This means that even when the gut luminal *S*.Tm population supports a high MOI in close contact with the mucosa for several hours (Fig 5A–5C), cooperative *S*.Tm entry does not occur (Fig 5J). These data provide functional support for *S*.Tm discreet-invasion into the murine gut absorptive epithelium.

### *S*.Tm discreet-invasion preferentially occurs proximal to cellular junctions

**Fig 6:**
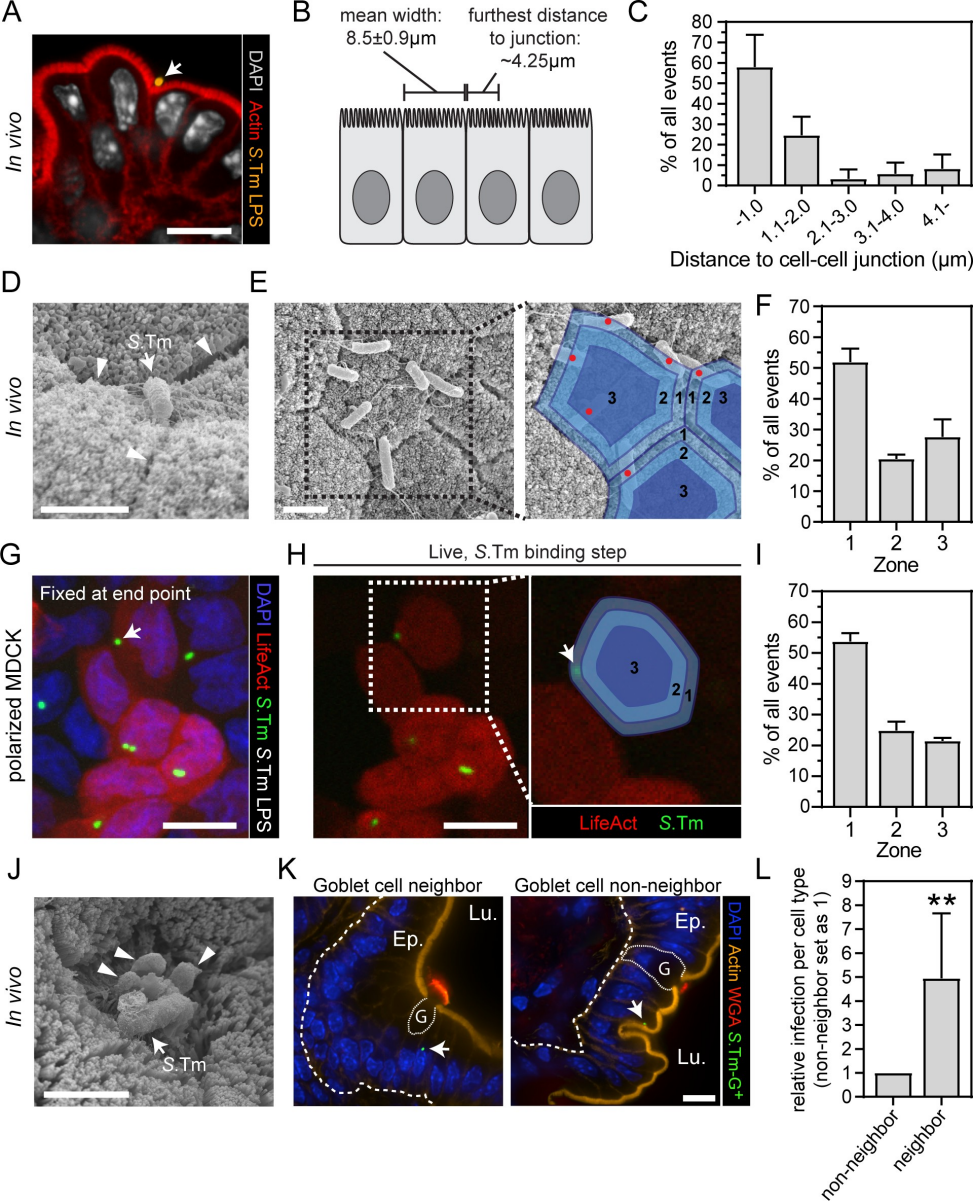
Cellular and subcellular *S*.Tm targeting preferences in the gut epithelium. (**A-F**) *S*.Tm preferentially binds the apicolateral region during first interactions with gut absorptive epithelial cells. (**A-C**) Wild-type mice were orally infected with *S*.Tm^*wt*^ for 9h. (**A**) Representative micrograph of the extensively washed cecal mucosa. Arrow head indicates a bound *S*.Tm. Scale bar: 10μm. (**B**) Experimentally determined dimensions of cecal absorptive epithelial cells (mean+/-SD). (**C**) Quantification of the distance between epithelial surface-bound *S*.Tm and the nearest cell–cell junction. Data in B-C correspond to mean+/-SD of replicate infections in four mice. (**D-E**) Representative SEM micrographs of the cecal epithelium of *Nlrc4*^*-/-*^ mice infected with *S*.Tm^*wt*^ for 12h. In D arrow heads indicate cell–cell junctions; arrow points to a bound *S*.Tm. In E, blue shading in the blow-up indicate zones used for quantification. Scale bars in D-E: 2μm. (**F**) Quantification of the distribution of epithelial surface-bound *S*.Tm^*Δ4*^ in the cecal epithelium of *Nlrc4*^*-/-*^ mice infected for 18h. Bars correspond to mean +/- range of replicate infections in two mice. (**G-I**) *S*.Tm preferentially invades polarized cultured epithelial cells at apicolateral sites. Polarized LifeAct-expressing MDCK cells (red) were infected with *S*.Tm^*wt*^/p*mCherry* (green) at MOI 50. (**G**) Representative maximum intensity projection micrograph of cells fixed at the end point (14min p.i.). (**H**) The first frame in the live imaging series where the indicated *S*.Tm (white arrow) could be found. Blue shading in the blow-up indicate zones used for quantification. Scale bars in G-H: 10μm. (**I**) Quantification of the distribution of epithelial cell invasion events in G-H. Bars correspond to mean +/- SD of four replicate MDCK infections. (**J-L**) Preferential *S*.Tm invasion of goblet-cell neighboring absorptive epithelial cells. (**J**) Representative SEM micrograph of the cecal epithelium of a wild-type mouse infected with *S*.Tm^*wt*^ for 8h. Arrow heads indicate extruding mucus from a goblet cell. arrow points to a bound *S*.Tm. Scale bar: 2μm. (**K-L**) Wild-type mice were orally infected with *S*.Tm^*wt*^*/*p*ssaG-GFP* for 6h. (**K**) Representative micrographs from confocal Z-stacks. Lu.—Lumen; Ep.—Epithelium; G—Goblet cell. White arrows point to *S*.Tm invasion foci. Scale bar: 10μm. (**L**) Quantification of the frequency of *S*.Tm invasion into goblet cell neighboring and non-neighboring epithelial cells. Bars correspond to mean +/- SD of replicate determinations in ten cecal tissue sections. Mann-Whitney U-test (**p<0.01).

While studying the impact of apical *S*.Tm^*wt*^ binding to the gut epithelium *in vivo*, we noted that bacteria frequently localized to the cell–cell junctional zones, separating individual epithelial cells (e.g. Fig 3H and 3L panel i, S4B Fig). These circumstantial observations prompted us to investigate if *S*.Tm discreet-invasion exhibits preference for specific apical locations. To study the surface-binding *S*.Tm population *in vivo* in isolation, luminal bacteria were removed by repeated gentle washing of infected tissue, prior to fixation and staining of the remaining adherent *S*.Tm^wt^ (Fig 6A). Again, we noted only modest perturbations of the local actin brush border proximal to attached *S*.Tm. Moreover, *S*.Tm surface binding exhibited a highly non-random pattern; ~80% of all bacteria could be found within a 2μm distance from the closest cell–cell junction (Fig 6B and 6C).

We adapted the procedure for SEM imaging of the gut epithelial surface from the luminal side (Fig 6D). To quantify the distribution of bound *S*.Tm, the surface of each epithelial cell was subdivided into three zones of equal area. Zone 1 covered the junction-proximal part, zone 2 the intermediate part, and zone 3 the mid part of the cell surface (Fig 6E). The center of each bound *S*.Tm was subsequently mapped onto these zones. To increase the number of bacteria that could be observed in this transient pre-invasion state, we infected mice with *S*.Tm^*Δ4*^, which lacks all the major TTSS-1 effectors, but remains competent for host cell binding. A marked enrichment of *S*.Tm binding was noted in zone 1, which carried a higher fraction of all bound bacteria than zone 2+3 combined (Fig 6F).

To test if not only binding, but also *S*.Tm invasion, exhibited preference for the apicolateral zone in polarized epithelial cell layers, we next performed a similar analysis in live polarized MDCK cells expressing LifeAct. Fixation and staining for *S*.Tm-LPS without permeabilization was used at the end point of the infection to focus the analysis on successful invasion events (Fig 6G). Each event was traced back to the moment of entry in the live series, and the apical host cell membrane subdivided into zones as above. In full agreement with results from the binding experiments *in vivo*, a majority of *S*.Tm invasion events mapped to zone 1 (Fig 6H and 6I). Based on these data, we conclude that an apicolateral surface region represents a hotspot for *S*.Tm binding and invasion of the polarized gut absorptive epithelium.

By contrast to homogeneous epithelial cell lines, the intact gut epithelium comprises multiple cell types in addition to absorptive epithelial cells. Of these, mucus-producing goblet cells make up ~12% of the total cell-count in the murine cecum. In the SEM analysis, we frequently observed that epithelium-adherent *S*.Tm localized close to the junctional zones between an absorptive epithelial cell and its goblet cell neighbor (Fig 6J). The *S*.Tm/p*ssaG-GFP* reporter strain was therefore again used to examine the neighborhoods of epithelial cells targeted by *S*.Tm invasion. The results revealed a ~5-fold enrichment of *S*.Tm invasion events into goblet-cell-neighboring epithelial cells, as compared to goblet cell non-neighbors (Fig 6K and 6L). This points to a non-random targeting of *S*.Tm epithelial cell discreet-invasion both at the cellular (i.e. goblet-cell neighbors) and subcellular (junctional zone-proximal) level.

## Discussion

**Fig 7:**
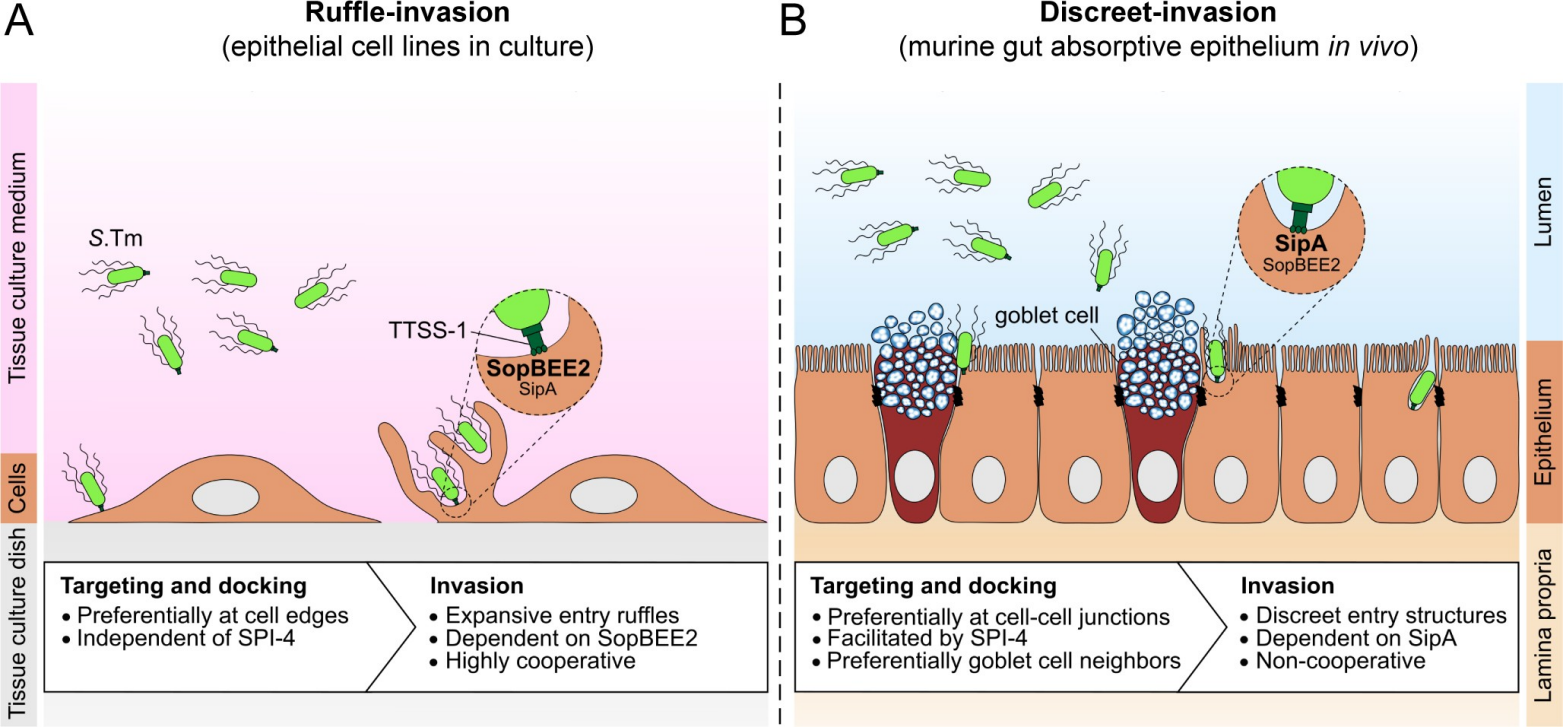
*S*.Tm can trigger ruffle-invasion or discreet-invasion depending on the host cell context. A conceptual model for *S*.Tm (**A**) ruffle-invasion of cultured cell lines and (**B**) discreet-invasion of gut absorptive epithelial cells *in vivo* in mice.

Tissue culture studies have established that *S*.Tm invades epithelial cells through TTSS-1 and SopBEE2-driven large membrane ruffles [3,4]. We have confirmed these findings across cell culture models from diverse species and found that ruffle-invasion accounts for 100% of the *S*.Tm entry events. Such ruffles are characterized by a mix of actin meshwork-containing lamellipodia and spike-like filopodial protrusions, induced by parallel activation of several actin regulatory Rho and Arf GTPases (e.g. Rac1, Cdc42, Arf1; [4]). The large size and dynamic nature of *S*.Tm-induced ruffles, combined with bacterial near-surface swimming, also accounts for the prevalent cooperative uptake of bystander bacteria [5,18] (Fig 7A). Importantly however, we here argue that *S*.Tm invasion of absorptive epithelial cells in the mouse gut proceeds by discreet-invasion, a process with distinct molecular and morphological properties (Fig 7B). Specifically, discreet-invasion of the murine gut absorptive epithelium i) is facilitated by the SPI-4 adhesin system, ii) depends strongly on the primordial TTSS-1 effector SipA, iii) exhibits only a moderate dependence on the ruffle-inducers SopBEE2, iv) does not promote cooperative entry, v) drives formation of discreet and transient entry structures distinct from prototypical ruffles, vi) preferably targets the apicolateral membrane at cellular junctions, and vii) results in preferential invasion of goblet-cell neighboring epithelial cells. All of these features contrast to observations of the ruffle-invasion process in epithelial cell lines (Fig 7A and 7B). Moreover, discreet-invasion also appears distinct from the TTSS-1-independent entry mechanism(s) that have been described e.g. in cultured fibroblasts [63,64], since discreet-invasion of the gut epithelium requires both TTSS-1 and SipA.

Differences in epithelial cell polarity may contribute to the observed differences in *S*.Tm invasion mechanisms. When epithelial cell lines were grown as a semi-polarized/polarized cell layers, the size of *S*.Tm-induced ruffles decreased in comparison to non-polarized counterparts. By time-lapse imaging of polarized MDCK cells we also detected a ~5% fraction of *S*.Tm^wt^ invasion events that proceeded in the absence of visible actin ruffles. Still, bacterial entry through ruffling appears commonplace in this host cell context (this study and [27,65,66]). Previous work also showed that *S*.Tm invasion of polarized epithelial cell lines involves cooperative entry [65], similar to in non-polarized cell lines [5,18,19,26], while *S*.Tm invasion of the mouse gut absorptive epithelium occurs in the absence of cooperativity (this study). Furthermore, we observed only a modest SipA-dependent invasion phenotype in polarized epithelial cell lines cells (~1.5-3-fold attenuation), compared to the ≥100-fold invasion defect in the gut epithelium *in vivo*. Based on this, we propose that polarized cell lines morphologically resemble columnar *in vivo* epithelia, but may retain (or acquire in culture) some immature properties that still overemphasize ruffling responses elicited by SopBEE2. It is here noteworthy that cellular transformation often results in overexpression of cytoskeletal regulators, including Rho-GTPases [67], which are common targets for these *S*.Tm effectors.

Early electron microscopy studies of *S*.Tm infection in starved and opium-treated guinea pigs [40], or in Peyer’s patches of calves, mice, and pigs [41–44], revealed *S*.Tm invasion of both M-cells, absorptive epithelial cells, and goblet cells. Peyer’s patch M-cells exhibited membrane ruffling with lamellipodial features in response to *S*.Tm [41,42]. In pig absorptive epithelial cells, elongated and distorted microvilli could be found at entry sites, i.e. in line with our results herein [44]. However, some examples of more pronounced cell surface perturbations were also noted in calves [41,43]. Hence, it appears plausible that the mechanism of *S*.Tm epithelial cell invasion *in vivo* may vary along the spectrum from “ruffle-invasion” to “discreet-invasion”, as a consequence of the epithelial cell type afflicted, the host species, the developmental stage of the epithelium and/or the effector repertoire of the *S*.Tm strain. In gut absorptive epithelial cells of adult mice infected with *S*.Tm SL1344, discreet-invasion appears to constitute the norm (Fig 7).

The prominent role of SipA during epithelial cell invasion in mice was unexpected, and contrasts to findings in cell lines ([25,27]; this study). On the molecular level, SipA has been shown to bind directly to actin, stabilize and bundle actin filaments, and in combination with SipC stimulate actin nucleation [22,25,68]. In cellular extracts, addition of SipA also prevents actin filament disassembly by ADF/Cofillin or Gelsolin [24]. Villin, another actin-binding and severing protein, is highly enriched in the brush border of intestinal epithelial cells. Knockdown of Villin in a polarized epithelial cell line attenuated *S*.Tm invasion and *Villin*^*-/-*^ mice showed a blunted mucosal tissue response to *S*.Tm infection [66]. It appears plausible that one or several of the actin-supporting functions of SipA can explain the discreet outgrowth of microvilli around invading *S*.Tm *in vivo*, as noted in our SEM analysis. Furthermore, a screen for host cell factors involved in SipA-driven HeLa cell invasion identified members of the SPIRE family of actin nucleation factors [26]. SPIRE2 is also highly expressed in gut epithelial cells [69]. It, however, seems less likely that SPIRE(s) act directly downstream of SipA, since SPIRE1/2 ablation resulted in a general decrease in invasiveness also for *S*.Tm strains invading by other means [26]. Nevertheless, the profound impact of SipA on epithelial cell invasion in adult (this study), as well as neonate mice [45], warrants further studies of how the biochemical activities of this effector are integrated in the *in vivo* context.

Previous work has demonstrated important role(s) for SipA in shaping the *S*.Tm niche in the vacuole or cytosol after host cell entry [45,52–54]. Our data do not refute that SipA can act in this fashion also in the intact epithelium of adult mice. However, several lines of evidence still support a predominant effect of SipA during the epithelial cell invasion step *in vivo*. First, our approach with multiple methods confirmed that our results do not depend on the use of specific fluorescent reporters. Second, microscopy of washed tissue showed *S*.Tm^*ΔsipA*^ to be enriched on the epithelial surface similar to *S*.Tm^*wt*^, but to be incapable of accumulating inside the mucosal epithelium. Third, *S*.Tm replicative foci are on average small (mean ~2 bacteria) and cytosolic replication rare in the epithelium of adult wild-type mice [30,70], where we noted ≥100-fold reduced bacterial loads upon SipA deletion (this study). Consequently, total *S*.Tm loads in the murine gut epithelium depend strongly on the *de novo S*.Tm invasion rate, which in turn depends on the TTSS-1 effector SipA.

Moreover, we found that *S*.Tm targets absorptive epithelial cells preferentially proximal to cell–cell junctions *in vivo*. Near-surface swimming allows *S*.Tm to scan along a plastic dish or host cellular surface, resulting in preferential trapping and docking at the base of physical obstacles (e.g. preexisting membrane ruffles) [5]. The junctions between individual gut epithelial cells, and between goblet cells and their neighboring enterocytes, constitute interruptions of an otherwise homogenous surface. Hence, the observed *S*.Tm targeting preference may stem from near-surface swimming along the epithelium surface and subsequent trapping at cell–cell junctional sites of unevenness. Direct testing of this hypothesis will require further development of *in vivo* imaging technology. We have by such technology recently shown that *S*.Tm exhibits “near-mucus surface swimming” in the murine gut [62], which can explain the high likelihood of goblet cell neighboring epithelial cells being targeted for *S*.Tm discreet-invasion.

From an evolutionary standpoint, SPI-1 acquisition represented a significant leap in the evolution of a harmless commensal *E*. *coli*-like bacterium towards modern pathogenic *Salmonella* spp. It is here relevant to note that TTSS-1 and SipA, but not SopBEE2, are encoded directly within SPI-1. Hence, TTSS-1 and SipA can be considered the primordial virulence arsenal that enabled *Salmonella* spp. to invade the host's gut epithelium. The genes for the ruffle-inducers were acquired as separate elements, SopB located within SPI-5 [71], SopE on a bacteriophage found only in some serovars [72], and SopE2 on a phage remnant [9]. Despite their invasion-promoting potential in cultured cell lines, the functions of SopB, SopE, and SopE2 during gut infection remain a matter of debate. These effectors may generally enhance invasive behavior, drive ruffle-invasion into specific host cell types, or promote absorptive epithelial cell invasion in particular host species or developmental stages. Alternatively, acquisition of SopBEE2 has primarily served the purpose of driving gut inflammation through activation of pro-inflammatory signaling in the mucosa [35,38,39,73–75]. In either case, also without SopBEE2, TTSS-1 combined with SipA constitutes a remarkably efficient minimal system for *S*.Tm discreet-invasion of absorptive gut epithelial cells in their native niche. The present study motivates continued efforts towards uncovering the molecular facets of this *in vivo* invasion mechanism.

## Materials and methods

### Ethics statement

All animal experiments were performed in accordance to the Swiss Federal Government guidelines in the animal experimentation law (SR 455.163 TVV). The protocols used were approved by the Cantonal Veterinary Office of the canton Zürich, Switzerland (Kantonales Veterinäramt ZH licenses 223/2010, 222/2013, and 193/2016).

### Bacterial strains, plasmids and culture conditions

All *S*.Tm strains used in this study were isogenic derivatives of SL1344 [76]. Strains and bacterial plasmids are detailed in S1 Table. For infections, *S*.Tm strains were grown in LB broth/0.3M NaCl, supplemented with 50μg/mL streptomycin (AppliChem), 50μg/mL ampicillin (AppliChem), or 12.5μg/mL chloramphenicol (Sigma Aldrich) for 12h at 37°C in a rotating wheel incubator. Cultures were diluted 1:20 in the same broth w/o antibiotics and cultured for an additional 4h at 37°C. For epithelial cell line infections, the inoculum was diluted in tissue culture medium to achieve the indicated MOI. For mouse infections, the inoculum was washed in sterile phosphate-buffered saline pH 7.4 (PBS; Gibco or Amimed) and reconstituted in PBS to a concentration of ~10^9^ CFUs/ml.

### Epithelial cell line culture and infections

HeLa (CCL-2; ATCC), MDCK (subclone Pf´ [37]; kind gift from Michael Hensel), and MDCK-LifeAct-GFP transfectants cells were maintained in DMEM (Gibco) supplemented with 10% heat-inactivated fetal calf serum (FCS) (Invitrogen). m-ICc12 cells [51] were cultured in DMEM/F12 (Invitrogen) supplemented with 5μg/ml insulin (Invitrogen), 50nM dexamethasone (Sigma Aldrich), 60nM sodium selenite (Sigma Aldrich), 5μg/ml bovine apo-transferrin (Sigma Aldrich), 1nM triiodothyronine (Sigma Aldrich), 60ng/ml EGF (Sigma Aldrich), 2mM glutamine (Invitrogen), 12.5mM D-glucose (Sigma Aldrich), 20mM hepes (Gibco), and 2% heat-inactivated FCS. Caco-2 C2Bbe1 (CRL-2102; ATCC) cells were maintained in DMEM supplemented with 10μg/ml human Transferrin (Sigma Aldrich), and 10% heat-inactivated FCS. For growth atop Transwell inserts, Caco-2 C2Bbe1 cells were seeded out in DMEM supplemented with 1:1000 Mito+ serum extender (Corning), and the medium exchanged at 24h post-seeding for Enterocyte Differentiation Medium (Corning) with Mito+ serum extender. m-ICc12 cells were grown atop Transwell inserts in their regular culture medium. Transwells were pre-coated with Collagen-1 (~30μg/cm2; rat-tail Collagen-1; Corning) prior to cell seeding. The status of monolayers atop Transwell inserts was followed by TEER measurement (EVOM^2^ voltohmmeter with STX2 chopsticks electrode; World Precision Instruments). For live imaging, HeLa cells were transiently transfected with LifeAct-mCherry (mCherry-Lifeact-7; kind gift from Michael Davidson; addgene construct #54491) using Lipofectamine^TM^ 2000 (Thermo Fisher) according to the manufacturer’s protocol. All culture media used was supplemented with 50μg/mL streptomycin sulphate or with PenStrep (Gibco), but antibiotics were omitted in preparation for infection. Cell lines were kept at 37°C in a CO_2_-buffered incubator and infected by adding a given *S*.Tm strain for the indicated time. For enumeration of *S*.Tm invasion efficiency by plating, infected cells were washed with fresh culture medium, incubated with culture medium containing 200μg/ml gentamicin (AppliChem) ≥40min, washed with PBS, lysed in 0.1% sodium deoxycholate (Sigma Aldrich), and serial dilutions of the lysates plated on LB agar with appropriate antibiotics.

### Mice and *in vivo* infections

Mice were bred and kept in individually ventilated cages under specific pathogen free conditions (RCHCI and EPIC facilities, ETH Zürich). Wild-type C57BL/6 mice were originally from Charles River, *Rag1*^*-/-*^ (B6.129S7-Rag1tm1Mom/J) from Jackson Laboratory, and *Nlrc4*^*-/-*^ mice have been described elsewhere [57]. *S*.Tm infections were performed as detailed in [33]. In brief, ~8–12 week old mice were treated with 25mg streptomycin sulphate per oral gavage. This step is required to suppress gut microbiota colonization resistance and thereby permit luminal expansion of *S*.Tm. 24h later, mice were infected per oral gavage with 5x10^7^ CFUs of the indicated *S*.Tm strain. Bacterial loads in gut lumen content and organs were monitored by plating homogenized samples on MacConkey agar (Oxoid) with 50μg/ml streptomycin. Plating of cecum tissue was done subsequent to a 30min incubation in PBS/400μg/ml gentamicin, six rigorous washes in excess PBS, and tissue homogenization. For histopathology scoring, cecum tissue was frozen in optimum cutting temperature medium (OCT; Tissue-Tek). 5μm cross-sections were air dried and stained with hematoxylin and eosin. Histopathology scoring was done blindly as described [33]. Briefly, a score was assigned based on the degree of submucosal edema, polymorphonuclear leukocyte infiltration, goblet cell numbers, and epithelial damage. The possible scores range from 0 (uninflamed) to 13 (maximum inflammation).

### Fluorescence microscopy of fixed cell lines and tissues

Uninfected or *S*.Tm-infected epithelial cell lines grown as indicated were fixed in 2–4% room-temperatured paraformaldehyde (PFA)/4% sucrose or in ice-cold methanol (for tubulin staining). Cells were permeabilized with PBS/0.1% TritonX-100 (Sigma Aldrich) or PBS/0.1% saponin (Calbiochem), and unspecific binding blocked in PBS/10%FCS. After staining with the indicated antibodies (detailed below), cells were mounted with Mowiol (Calbiochem) and imaged the following day. Mouse cecum tissue (or other intestinal segment if indicated) was fixed in 4% PFA, saturated in PBS/20% sucrose, embedded in OCT, flash-frozen, and stored at −80°C. In cases where luminal bacteria were removed, the intestinal segment was opened longitudinally and washed extensively in PBS before fixation and embedding. 10–20μm cryosections were placed on glass slides, air dried, rehydrated in PBS, permeabilized with PBS/0.5% Triton X-100, and blocked in PBS/10% normal goat serum (NGS; Vector Labs). After immunostaining, the samples were mounted with Mowiol and imaged the following day. Antibodies and staining reagents used in this study were α-EpCAM/CD326 (G8.8, Biolegend), α-gp135 (3F2/D8-s; DSHB), α-ICAM-1/CD54 (clone 3E2, Becton Dickinson), α-M45 (purified from hybridoma), α-S.Tm LPS (O-antigen group B factor 4–5, Difco), α-tubulin (B5-1-2; Sigma Aldrich), α-hamster-Cy3 (Jackson), α-mouse-Cy3 (Jackson), α-mouse-Cy5 (Jackson), α-rabbit-Cy3 (Jackson), α-rabbit-Cy5 (Jackson), AlexaFluor647-conjugated wheat-germ agglutinin (Molecular Probes), TRITC-conjugated Phalloidin (Fluoprobes), AlexaFluor488 or 647-conjugated phalloidins (Molecular Probes), and DAPI (Sigma Aldrich). Automated microscopy analysis of invasion efficiency in epithelial cell lines was performed as described in detail elsewhere [77]. For confocal microscopy, we used a Zeiss Axiovert 200m microscope with 10X–100X objectives, a spinning disc module (Visitron), and two Evolve 512 EMCCD cameras (Photometrics), or a Nikon Eclipse Ti2 microscope core fitted with 10-100X objectives, a X-Light-V2-LFOV spinning disc module (Crest), and a Prime 95B 25mm camera (Photometrics). Images were captured and processed using the Visiview software (Visitron) and/or Image J ×64. For quantification of *S*.Tm/p*ssaG-GFP* bacteria in mouse intestinal epithelial tissue, 20μm cross-sections stained with ICAM-1, phalloidin, and DAPI, were imaged at 400X–1,000X, and intracellular (GFP+) *S*.Tm were enumerated blindly in six to nine non-consecutive sections/mouse. Data represent averages/section.

### Live microscopy of infected cell lines

15.000 LifeAct-expressing HeLa (LifeAct-mCherry) or MDCK (LifeAct-GFP) cells were seeded into glass-bottomed culture dishes (ibidi) in culture medium 24 hours prior to the experiment. To induce polarization of MDCK cells, 25.000 cells were seeded into culture dishes and incubated for 7 days, with medium exchange after 3, 6, and 7 days. Short-term live microscopy was performed in HBSS (Gibco)/10% FCS/20mM Hepes. Cells were infected with *S*.Tm^*wt*^/p*mCherry* or *S*.Tm^*wt*^/p*GFP* (MOI ~50) or with *S*.Tm^*ΔsopBEE2*^/p*mCherry* or *S*.Tm^*ΔsopBEE2*^/p*GFP* (MOI ~500). Movies were acquired on a Nikon Eclipse T1 inverse microscope equipped with a Yokogawa CSU-W1-T2 spinning-disk confocal unit and two EMCCD ixon888 cameras, using a 20x objective (non-polarized cells; PLAN Apochromat, NA 0.75) or a 60x oil objective (polarized MDCK; PLAN Apochromat, NA 1.4). Movies were acquired for 14 min—40 min and time-lapse data were reconstructed and analyzed in Image J ×64. Subsequent to live cell imaging, infected cells were washed with HBSS/10% FCS, fixed with 4% PFA, washed with 4% sucrose, saturated in 20% sucrose, and incubated in a PBS/3% bovine serum albumin/3% sucrose blocking buffer. Extracellular bacteria were labeled using α-*S*.Tm LPS and α-rabbit-Cy5. Cells were subsequently permeabilized in 0.1% Triton X-100 and stained with DAPI. Image acquisition of the same areas previously recorded by live microscopy was performed using a 100X oil objective (PLAN Apochromat, NA 1.49; non-polarized cells) or a 60X objective (PLAN Apochromat, NA 1.4; polarized MDCK cells). Image z-stacks were collected using the VisiVIEW software and further analyzed in Fiji. For consistency, the LifeAct signal is presented as red and *S*.Tm as green in all figure panels.

### Cryo-electron microscopy and cryo-electron tomography

EM finder grids (gold NH2 R2/2, Quantifoil) were sterilized under UV light and then glow discharged. Grids were placed on the bottom of wells in a 12-well plate (Nunc, Thermo Fisher) and equilibrated in DMEM. Subsequently, 30.000 HeLa cells were seeded into each well and incubated overnight. Cells were infected with *S*.Tm^*wt*^ and *S*.Tm^*ΔsopBEE2*^ as indicated. Plunge freezing was performed according to [78]. Briefly, grids were removed from the wells using tweezers, these subsequently mounted in a Vitrobot (Thermo Fisher) and the grids blotted from the backside by installing a Teflon sheet on one of the blotting pads. Grids were plunge-frozen in liquid ethane-propane (37%/63%) and stored in liquid nitrogen as described [79]. Infected cells were examined by cryo-electron microscopy (cryoEM) and cryo-electron tomography (cryoET) as detailed in [78]. Images were recorded on a Tecnai Polara TEM (Thermo Fisher) equipped with post-column GIF 2002 imaging filter and a K2 Summit direct electron detector (Gatan), or on a Titan Krios TEM (Thermo Fisher) equipped with a Quantum LS imaging filter and a K2 Summit. Both microscopes were operated at 300kV and the imaging filters were set to 20 eV slit width. The pixel size at the specimen level ranged between 4.29–5.95Å. Tilt series covered an angular range from -60° to +60° with 1° increments and -8 μm defocus. The total dose of a tilt series was 120 e^-^/Å^2^. Tilt series and 2D projection images were acquired automatically using UCSF Tomo [80] or SerialEM [81]. Three-dimensional reconstructions and segmentations were generated using the IMOD program suite [82].

### Field emission scanning electron microscopy

Infected mouse cecal tissue or HeLa cells were fixed in 2.5% glutaraldehyde (Polyscience). After washing in Krebs-Ringer buffer, 1% OsO_4_ (Polyscience) was used for post-fixation, or samples were washed in TE buffer (TRIS 10 mM, EDTA 1 mM, pH 7.0) without further osmification. HeLa cells were additionally incubated in 0.5% carbohydrazide and treated with 1% OsO_4_ for a second time. All samples were washed before dehydration in a graded series of acetone. Critical-point-drying was performed with liquid CO_2_ using an Autosamdri-931 (Tousimis or Bal-Tec CPD030). Thereafter, samples were mounted on aluminum SEM stubs, sputter coated with 5nm platinum/palladium or palladium/gold (Safematic CCU-010 or Bal-Tec SCD500). Samples were examined using a Zeiss Merlin Gemini II ultra-high resolution field emission scanning electron microscope at an acceleration voltage of 5 kV. Images were captured and analyzed with Zeiss SmartSEM and Image J ×64.

### Barcoded consortium infections

Barcoded *S*.Tm strains are detailed in S1 Table. Strains were grown in LB/0.3M NaCl/12.5μg/ml chloramphenicol for 12h at 37°C, diluted 1:20 in LB/0.3M NaCl and cultured for another 4h at 37°C. A 1:1:1:1:1:1:1 mix of all seven strains (or a 1:1:1:1:1:1 mix of a six strain consortium for S3E Fig) was used as inoculum. Cell lines were seeded in wells or atop Transwell inserts as indicated and infected with the mixed consortium inoculum for 7-20min at low MOI (i.e. 0.2–2; to limit the impact of cooperative invasion). Extracellular *S*.Tm were killed by 200μg/ml gentamicin for 1h and cells were washed and lysed in 0,1% sodium deoxycholate. The diluted inoculum and the retrieved intracellular bacterial population were enriched for 16h in tubes with LB (+/-12.5μg/ml chloramphenicol) at 37°C. For mouse infections, the mixed inoculum was resuspended in PBS and used to infect sm-pretreated *Nlrc4*^*-/-*^ mice with 5×10^7^ total CFUs per oral gavage. Animals were sacrificed ~18h p.i. The cecum was opened longitudinally, washed extensively in PBS, and treated with 400μg/ml gentamicin for 30min. The tissue was washed an additional nine times in PBS and homogenized in a TissueLyzer (Qiagen). *S*.Tm populations in cecum content and cecal tissue lysate were enriched in parallel in LB/12.5μg/ml chloramphenicol at 37°C. Total bacterial loads in cecum content and tissue was evaluated by plating a subfraction of the homogenates on MacConkey agar with 50μg/ml streptomycin. Genomic DNA from enrichment cultures was extracted using the GenElute^™^ Bacterial Genomic DNA Kit (Sigma Aldrich) and quantitative PCR analysis performed on 9ng of total genomic DNA, using the Maxima SYBR Green/ROX qPCR Master Mix (2X) (Thermo Scientific). Primers are detailed in S2 Table. The relative abundance of each barcoded strain was normalized to the corresponding mixed inoculum for HeLa cell infections, or to the corresponding cecum content for animal infections.

### Western blot

The indicated S. Tm strains were grown overnight in LB/0.3M NaCl/50μg/ml streptomycin sulphate, sub-cultured 1:20 in 3ml LB/0.3M NaCl for 4h. S.Tm were harvested, boiled in SDS-sample buffer, and particulate debris was spun down. Samples were run on 15% SDS-PAGE gels at 90V, 120min and subsequently transferred onto nitrocellulose filter (Protran BA 85, GE Healthcare). Filters were washed in PBS-Tween20 (National diagnostics) and blocked with PBS-Tween/5% milk powder. The indicated proteins were detected with primary antibodies α-M45 (purified from hybridoma), α-SipA (purified from ascites), α-σ70 (Abcam), and an HRP-conjugated rabbit anti-mouse secondary antibody. Signals were revealed with the ECL chemiluminescence detection reagent (Amersham) onto SuperRX Fuji medical x-ray film (Fujifilm corporation), using a Fujifilm FPM 800A developer.

### Statistical analysis

Where applicable, statistical significance was assessed by the Mann—Whitney U-test, the Kruskal—Wallis with Dunn’s post-test, or the one-way ANOVA with Dunnett´s multiple comparisons test, as indicated in the figure legends.

## Supporting information

S1 Fig (supporting data for Fig 1). (**A-C**) Invasion efficiency of *S*.Tm^*wt*^ and *S*.Tm^*Δspi-4*^ p*ssaG-GFP* reporter strains in the indicated epithelial cell lines, infected for 20min, and analyzed at 4h p.i. by automated microscopy. Data points represent mean +/- range of two to three replicate infections. (**D**) m-ICc12 cells were infected with the indicated strains at MOI 62.5 for 20min. Quantification of intracellular bacteria after gentamycin treatment. Bars represent mean +/- SD of six replicate infections. One-way ANOVA with Dunnett´s test (n.s., not significant; **p<0.01). (**E**) *S*.Tm CFU counts in cecum content of three C57BL/6 wild-type mice infected with *S*.Tm^*wt*^ for 12h. (**F**) Quantification of intraepithelial *S*.Tm per 20μm section across the entire length of the cecum in the three mice described in E. Note that *S*.Tm invasion events distribute evenly across the cecum length. (**G-I**) Cell-type distribution of *S*.Tm invasion events during early infection. Wild-type (G-H) and *Nlrc4*^*-/-*^ (I) mice were orally infected with *S*.Tm^*wt*^ for 12h and 18h, respectively. (**G**) Representative micrographs of the cecal mucosa. Lu.—Lumen; Ep.—Epithelium; L.p.—Lamina propria. White arrow heads indicate S.Tm invasion foci. Scale bar: 20μm. (**H-I**) Quantification of the cell type distribution of *S*.Tm invasion events into the cecal mucosa of (H) wild-type and (I) *Nlrc4*^*-/-*^ mice. EC–absorptive epithelial cell; GC–goblet cell; L.p.–lamina propria cell type(s). Bars correspond to mean +/- SD of replicate infections in four wild-type and three *Nlrc4*^*-/-*^ mice, respectively. Note that early *S*.Tm invasion events predominantly localize to absorptive epithelial cells. (**J-L**) Impact of TTSS-1 and SPI-4 on *S*.Tm gut absorptive epithelial cell invasion in mice. Wild-type mice were orally infected with the indicated *S*.Tm/p*ssaG-GFP* strains for 8-12h. (**J**) *S*.Tm CFU counts in cecum content. (**K**) Representative micrographs of the cecal mucosa. Lu.—Lumen; Ep.—Epithelium; L.p.—Lamina propria. White arrow heads indicate intraepithelial *S*.Tm. Scale bar: 10μm. (**L**) Quantification of intraepithelial *S*.Tm per 20μm section. In J and L each data point corresponds to one animal. Kruskal-Wallis with Dunn’s post-test (n.s., not significant; **p<0.01, ***p<0.001). (**M**) Western blot of SipA protein levels upon plasmid complementation of the indicated *S*.Tm strain. (**N-O**) Cecal histopathology at 12h p.i. with the indicated *S*.Tm strain (N) or combination of strains (O). Each data point corresponds to one animal. Mann-Whitney U-test in N (*p<0.05).
(TIF)

S2 Fig (supporting data for Fig 1). (**A-C**) Dependence on SipA for *S*.Tm invasion of subconfluent non-polarized (top panels) and confluent polarized (bottom panels) MDCK cells grown on cell culture plastic. (**A**) Representative micrographs from confocal Z-stack imaging of MDCK cells grown in the two arrangements. Scale bars: 10μm. (**B**) Invasion efficiency of the indicated *S*.Tm/p*ssaG-GFP* reporter strains in MDCK cells grown in the two arrangements, infected for 20min over a range of MOIs, and analyzed at 4h p.i. by automated microscopy. Data are expressed as nr of intracellular S.Tm (i.e. nr of reporter GFP spots) per well. One experiment is shown; representative for three experiments. (**C**) Invasion efficiency of the indicated *S*.Tm strains in MDCK cells grown in the two arrangements, infected at MOI 62.5 for 20min, and analyzed by selective plating of intracellular bacteria. Shown are CFU data for nine replicate infections (circle symbols) pooled from experimentation on two separate occasions. Bars represent mean values. Mann-Whitney U-test (n.s., not significant; **p<0.01). (**D-G**) Dependence on SipA for *S*.Tm invasion of semi-polarized m-ICc12 and polarized Caco-2 C2Bbe1 cells grown atop Transwell inserts. (**D-E**) Transepithelial epithelial resistance (TEER) over time for (D) m-ICc12 and (E) Caco-2 C2Bbe1, seeded on Transwell inserts either as a subconfluent layer for 24h (triangles), or as a confluent layer for up to 72/96h (inverted triangles). See materials and methods for details on the growth conditions. Data points correspond to mean +/- SD of six replicate Transwell seedings. Note that the m-ICc12 cells only develop into a moderate TEER (semi-polarized) monolayer, while Caco-2 C2Bbe1 cells form a tight monolayer with high TEER. Longer incubation times then those presented did not significantly increase the TEER values further for either of the cell lines. (**F-G**) Invasion efficiency of the indicated *S*.Tm strains in (F) semi-polarized m-ICc12 cells and (G) polarized Caco-2 C2Bbe1 cells grown atop Transwell inserts (conditions at 72/96h end-point of confluent growth, indicated by the inverted triangle symbols in D-E). Cells were infected at MOI 62.5 for 7 or 20min, and analyzed by selective plating of intracellular bacteria. Shown are CFU data expressed as the percentage of the inoculum retrieved in the intracellular population. Each circle symbol corresponds to one replicate infection. Bars represent mean+/- SD. One-way ANOVA with Dunnett´s test (n.s., not significant; **p<0.01).
(TIF)

S3 Fig (supporting data for Fig 2). (**A**) Dependence on SipA for *S*.Tm absorptive epithelial cell invasion *in vivo*. Inflammasome-deficient (*Nlrc4*^*-/-*^) mice were orally infected with the indicated *S*.Tm/p*ssaG-GFP* strains for 18h. Graph shows quantification of the number of intraepithelial *S*.Tm foci per 20μm section (mice also analyzed for total intraepithelial *S*.Tm loads in Fig 2C). Each data point corresponds to one animal. Line at median. (**B-G**) Barcoded consortium infections of epithelial cell lines and *in vivo* in mice. (**B**) Relative abundance of the individual strains in the barcoded consortium inoculum. The pie chart depicts the average from seven replicate experiments, where the relative abundance of each strain was assessed by quantitative PCR after enrichment culture. Note that none of the strains in the consortium is significantly over/underrepresented in the inoculum. (**C-D**) Barcoded consortium infections of (C) m-ICc12 cells on plastic, and (D) polarized Caco-2 C2Bbe1 cells grown atop Transwell inserts. The cells were infected for 20min at a total MOI of 2, using the same seven strain barcoded consortium as in Fig 2F and S3B Fig. Bars correspond to mean +/- SD of six (C) or three (D) replicate infections (circle symbols). (**E**) Barcoded consortium infection of polarized Caco-2 C2Bbe1 cells grown atop Transwell inserts with a less complex consortium. The cells were infected for 7, 10 or 20min at a total MOI of 2, using a barcoded consortium containing six tagged strains; two *S*.Tm^*wt*^ (tag C and tag D), two *S*.Tm^*ΔsipA*^ (tag B and tag F), and two *S*.Tm^*Δ4*^ strains (tag E and tag G) (see S1 Table). The relative abundance for *S*.Tm^*wt*^, *S*.Tm^*ΔsipA*^, and *S*.Tm^*Δ4*^ was calculated based on the summed abundance of the two internal technical replicates for each strain. Data points correspond to mean +/- SD of three replicate infections with separately prepared consortia. In C-E, One-way ANOVA with Dunnett´s test (n.s., not significant; *p<0.05, ***p<0.001). (**F-G**) Total *S*.Tm CFU counts in cecum content (F) and washed cecal tissue (G) of *Nlrc4*^*-/-*^ mice infected with the seven strain barcoded consortium for ~18h (barcode quantification data presented in Fig 2F). Each data point corresponds to one animal. Line at median.
(TIF)

S4 Fig (supporting data for Fig 3). (**A-B**) Additional fluorescence micrographs of early S.Tm invasion into gut absorptive epithelial cells *in vivo* in mice. (**A**) Additional representative micrographs of the cecal epithelium in wild-type mice orally infected with *S*.Tm^*wt*^/p*ssaG-GFP* for 6h, as in Fig 3F. Blow-up shows magnification of boxed region. Lu.–Lumen; Ep.–Epithelium; G.–Goblet cell. White arrow indicates the apical actin brush border of an infected absorptive epithelial cell. Scale bar: 10μm. (**B**) Additional representative micrographs of a SopE-M45 positive focus in the cecal epithelium in *Rag1*^*-/-*^ mice orally infected with *S*.Tm^*wt*^/p*sopE-M45* for 8h, as in Fig 3H. Blow-up shows magnification of boxed region. Lu.—Lumen. White arrow indicates an M45-positive bacterial focus. Scale bar: 10μm.
(TIF)

S5 Fig (supporting data for Fig 3). (**A-C**) Additional SEM micrographs of *S*.Tm invasion into epithelial cell lines and the absorptive gut epithelium *in vivo* in mice. (**A**) SEM micrographs of the *S*.Tm^*wt*^ inoculum used in Fig 3J–3L. (**B**) Additional SEM micrographs of HeLa cells infected with *S*.Tm^*wt*^ for 6-10min at MOI 400, as in Fig 3J. (**C**) Additional SEM micrographs of the cecal epithelium in mice, either uninfected, or upon infection with *S*.Tm^*wt*^, as in Fig 3L. Scale bars indicated separately for each panel. Arrow heads point to *S*.Tm. For each micrograph containing a dashed white box, the panel directly to the right of it represents the same area at higher magnification.
(TIF)

S6 Fig (supporting data for Fig 4). (**A-C**) *S*.Tm^*wt*^ invades a polarized epithelial cell line predominantly, but not exclusively, through induction of visible actin ruffles. Polarized LifeAct-expressing MDCK cells (red) were infected with *S*.Tm^*wt*^/p*mCherry* (green) at MOI 50. (**A**) Micrograph of cells fixed at the end point (14min p.i.). ExC—extracellular bacterium; R—ruffle;? —ambiguous entry event followed in the live series. (**B**) Live imaging series preceding A. R—ruffle; No R—no ruffle (encircled). (**C**) Quantification of the presence/absence of visible actin ruffles at *S*.Tm^*wt*^ entry sites. N_tot_ = 524 invasion events analyzed. (**D-F**) *S*.Tm^*ΔsopBEE2*^ consistently invades a polarized epithelial cell line without triggering visible actin ruffles. Polarized LifeAct-expressing MDCK cells (red) were infected with *S*.Tm^*ΔsopBEE2*^/p*mCherry* (green) at MOI 500. (**D**) Micrograph of cells fixed at the end point (40min p.i.). ExC–extracellular bacterium;? —ambiguous entry event followed in the live series. (**E**) Live imaging series of boxed region preceding D. Arrow head indicates a ruffle-less entry event. (**F**) Quantification of the presence/absence of visible actin ruffles at *S*.Tm^*ΔsopBEE2*^ entry sites. N_tot_ = 24 invasion events analyzed. Scale bars: 10μm.
(TIF)

S1 Movie Live microscopy of fluorescent *S*.Tm^*wt*^ (green) invading LifeAct-expressing (red) non-polarized MDCK cells. White arrows indicate ruffles. Time is indicated in min:second format.
(MP4)

S2 Movie Live microscopy of fluorescent *S*.Tm^*wt*^ (green) invading LifeAct-expressing (red) HeLa cells. White arrows indicate ruffles. Time is indicated in min:second format.
(MP4)

S3 Movie Live microscopy of fluorescent *S*.Tm^*ΔsopBEE2*^ (green) invading LifeAct-expressing (red) non-polarized MDCK cells. White arrow head indicates an entry event with no visible ruffle. Time is indicated in min:second format.
(MP4)

S4 Movie Live microscopy of fluorescent *S*.Tm^*wt*^ (green) invading LifeAct-expressing (red) polarized MDCK cells. White arrow indicates a ruffle and arrow head indicates an entry event with no visible ruffle. Time is indicated in min:second format.
(MP4)

S5 Movie Live microscopy of fluorescent *S*.Tm^*ΔsopBEE2*^ (green) invading LifeAct-expressing (red) polarized MDCK cells. White arrow head indicates an entry event with no visible ruffle. Time is indicated in min:second format.
(MP4)

S6 Movie Cryo-tomogram of *S*.Tm^*wt*^ bound to a HeLa cell. (MP4)

S7 Movie Cryo-tomogram of *S*.Tm^*wt*^ invading a HeLa cell. (MP4)

S8 Movie Cryo-tomogram of *S*.Tm^*ΔsopBEE2*^ invading a HeLa cell. (MP4)

S1 Table Bacterial strains and plasmids used in this study. (DOCX)

S2 Table Genetic barcodes and PCR primers for detection, used in this study. (DOCX)
