# Supplementary figures and images for: Salmonella Typhimurium discreet-invasion of the murine gut absorptive epithelium
Source: PLoS Pathog. 2020 May 4;16(5):e1008503. doi: 10.1371/journal.ppat.1008503 (PMC7224572; doi:10.1371/journal.ppat.1008503)

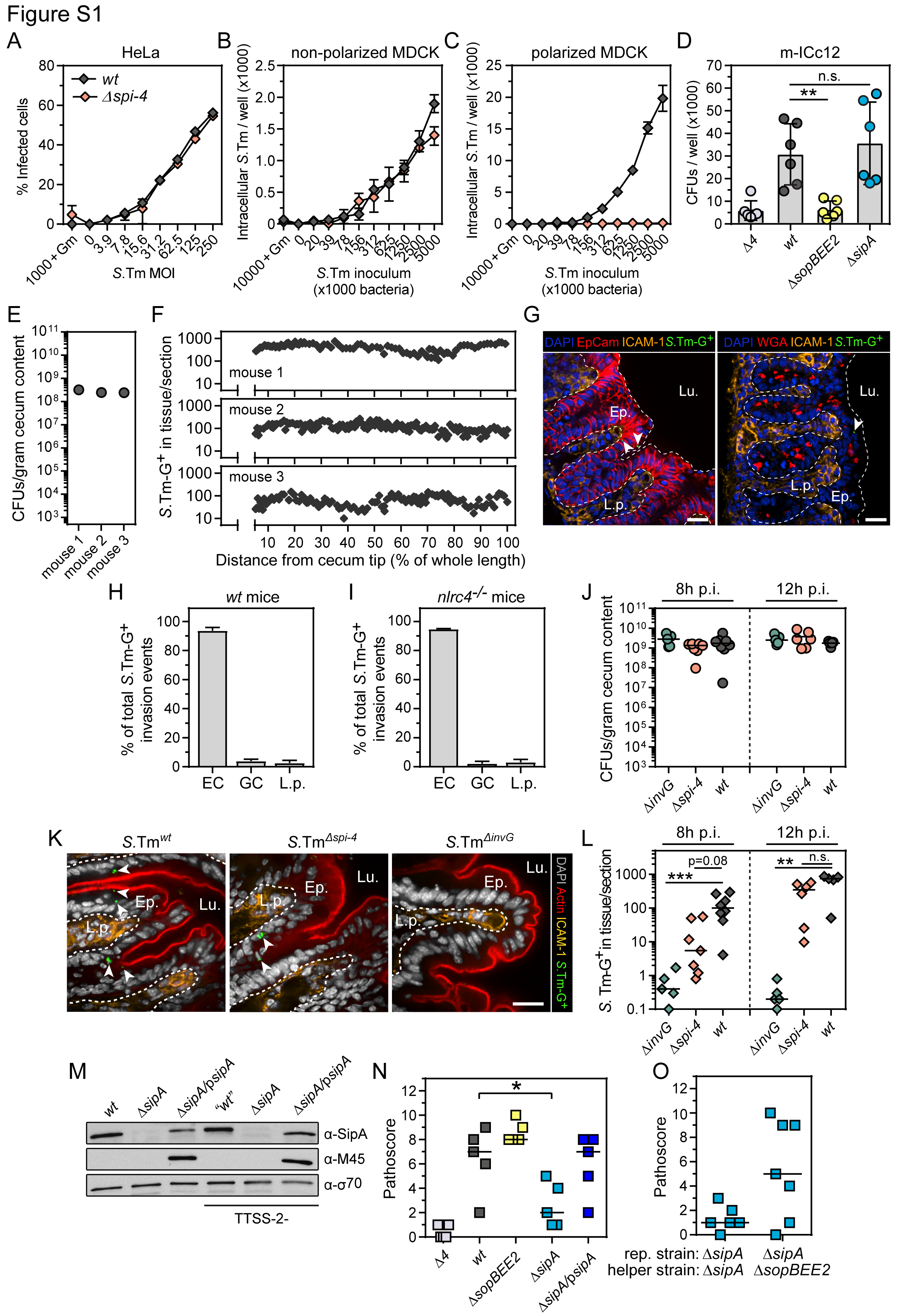

Supplement: S1 Fig — (A-C) Invasion efficiency of S.Tmwt and S.TmΔspi-4 pssaG-GFP reporter strains in the indicated epithelial cell lines, infected for 20min, and analyzed at 4h p.i. by automated microscopy. Data points represent mean +/- range of two to three replicate infections. (D) m-ICc12 cells were infected with the indicated strains at MOI 62.5 for 20min. Quantification of intracellular bacteria after gentamycin treatment. Bars represent mean +/- SD of six replicate infections. One-way ANOVA with Dunnett´s test (n.s., not significant; **p<0.01). (E) S.Tm CFU counts in cecum content of three C57BL/6 wild-type mice infected with S.Tmwt for 12h. (F) Quantification of intraepithelial S.Tm per 20μm section across the entire length of the cecum in the three mice described in E. Note that S.Tm invasion events distribute evenly across the cecum length. (G-I) Cell-type distribution of S.Tm invasion events during early infection. Wild-type (G-H) and Nlrc4-/- (I) mice were orally infected with S.Tmwt for 12h and 18h, respectively. (G) Representative micrographs of the cecal mucosa. Lu.—Lumen; Ep.—Epithelium; L.p.—Lamina propria. White arrow heads indicate S.Tm invasion foci. Scale bar: 20μm. (H-I) Quantification of the cell type distribution of S.Tm invasion events into the cecal mucosa of (H) wild-type and (I) Nlrc4-/- mice. EC–absorptive epithelial cell; GC–goblet cell; L.p.–lamina propria cell type(s). Bars correspond to mean +/- SD of replicate infections in four wild-type and three Nlrc4-/- mice, respectively. Note that early S.Tm invasion events predominantly localize to absorptive epithelial cells. (J-L) Impact of TTSS-1 and SPI-4 on S.Tm gut absorptive epithelial cell invasion in mice. Wild-type mice were orally infected with the indicated S.Tm/pssaG-GFP strains for 8-12h. (J) S.Tm CFU counts in cecum content. (K) Representative micrographs of the cecal mucosa. Lu.—Lumen; Ep.—Epithelium; L.p.—Lamina propria. White arrow heads indicate intraepithelial S.Tm. Scale bar: 10μm. (L) Quant [file ppat.1008503.s001.tif]

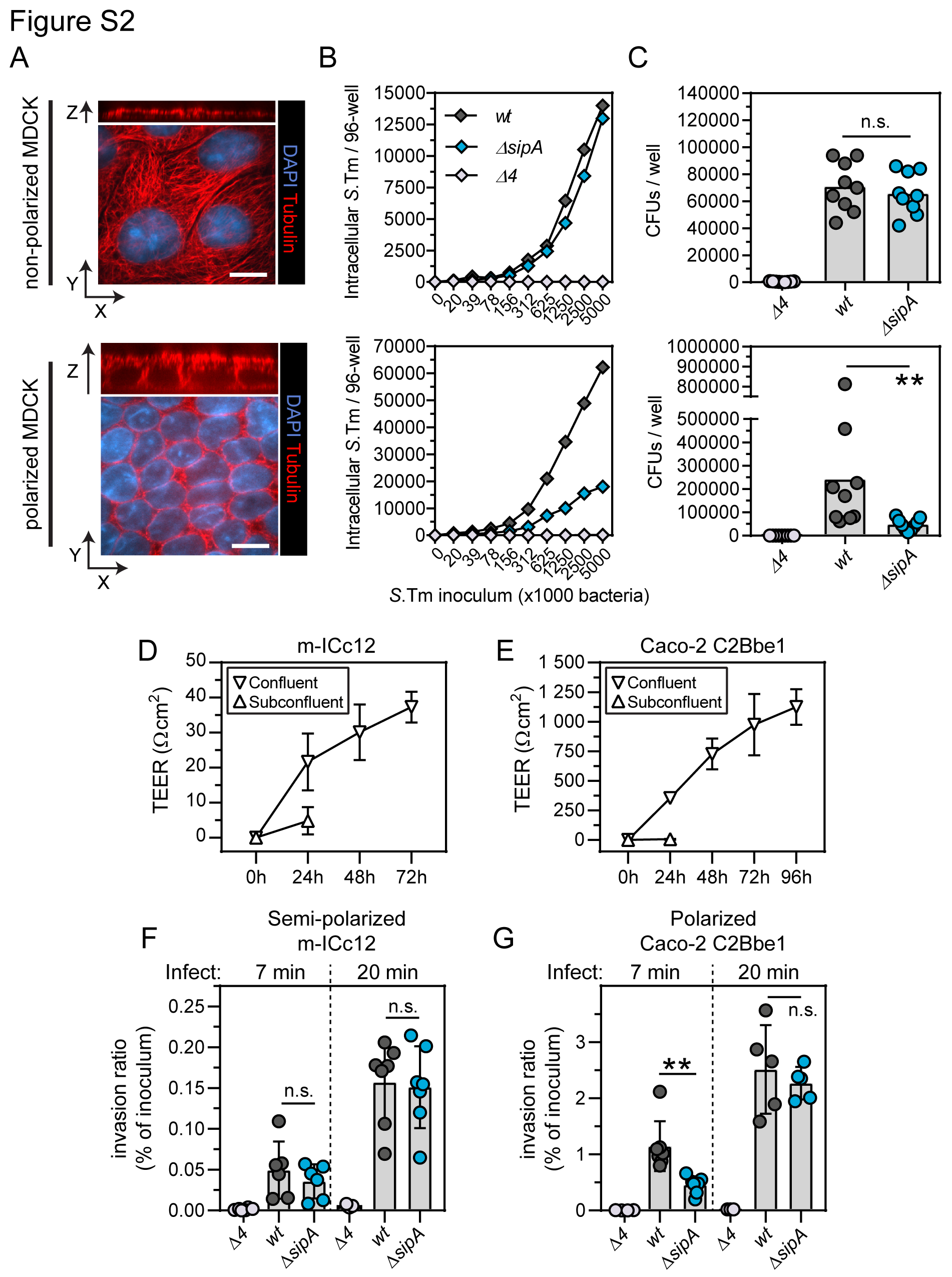

Supplement: S2 Fig — (A-C) Dependence on SipA for S.Tm invasion of subconfluent non-polarized (top panels) and confluent polarized (bottom panels) MDCK cells grown on cell culture plastic. (A) Representative micrographs from confocal Z-stack imaging of MDCK cells grown in the two arrangements. Scale bars: 10μm. (B) Invasion efficiency of the indicated S.Tm/pssaG-GFP reporter strains in MDCK cells grown in the two arrangements, infected for 20min over a range of MOIs, and analyzed at 4h p.i. by automated microscopy. Data are expressed as nr of intracellular S.Tm (i.e. nr of reporter GFP spots) per well. One experiment is shown; representative for three experiments. (C) Invasion efficiency of the indicated S.Tm strains in MDCK cells grown in the two arrangements, infected at MOI 62.5 for 20min, and analyzed by selective plating of intracellular bacteria. Shown are CFU data for nine replicate infections (circle symbols) pooled from experimentation on two separate occasions. Bars represent mean values. Mann-Whitney U-test (n.s., not significant; **p<0.01). (D-G) Dependence on SipA for S.Tm invasion of semi-polarized m-ICc12 and polarized Caco-2 C2Bbe1 cells grown atop Transwell inserts. (D-E) Transepithelial epithelial resistance (TEER) over time for (D) m-ICc12 and (E) Caco-2 C2Bbe1, seeded on Transwell inserts either as a subconfluent layer for 24h (triangles), or as a confluent layer for up to 72/96h (inverted triangles). See materials and methods for details on the growth conditions. Data points correspond to mean +/- SD of six replicate Transwell seedings. Note that the m-ICc12 cells only develop into a moderate TEER (semi-polarized) monolayer, while Caco-2 C2Bbe1 cells form a tight monolayer with high TEER. Longer incubation times then those presented did not significantly increase the TEER values further for either of the cell lines. (F-G) Invasion efficiency of the indicated S.Tm strains in (F) semi-polarized m-ICc12 cells and (G) polarized Caco-2 C2Bbe1 cells grown atop Transwell i [file ppat.1008503.s002.tif]

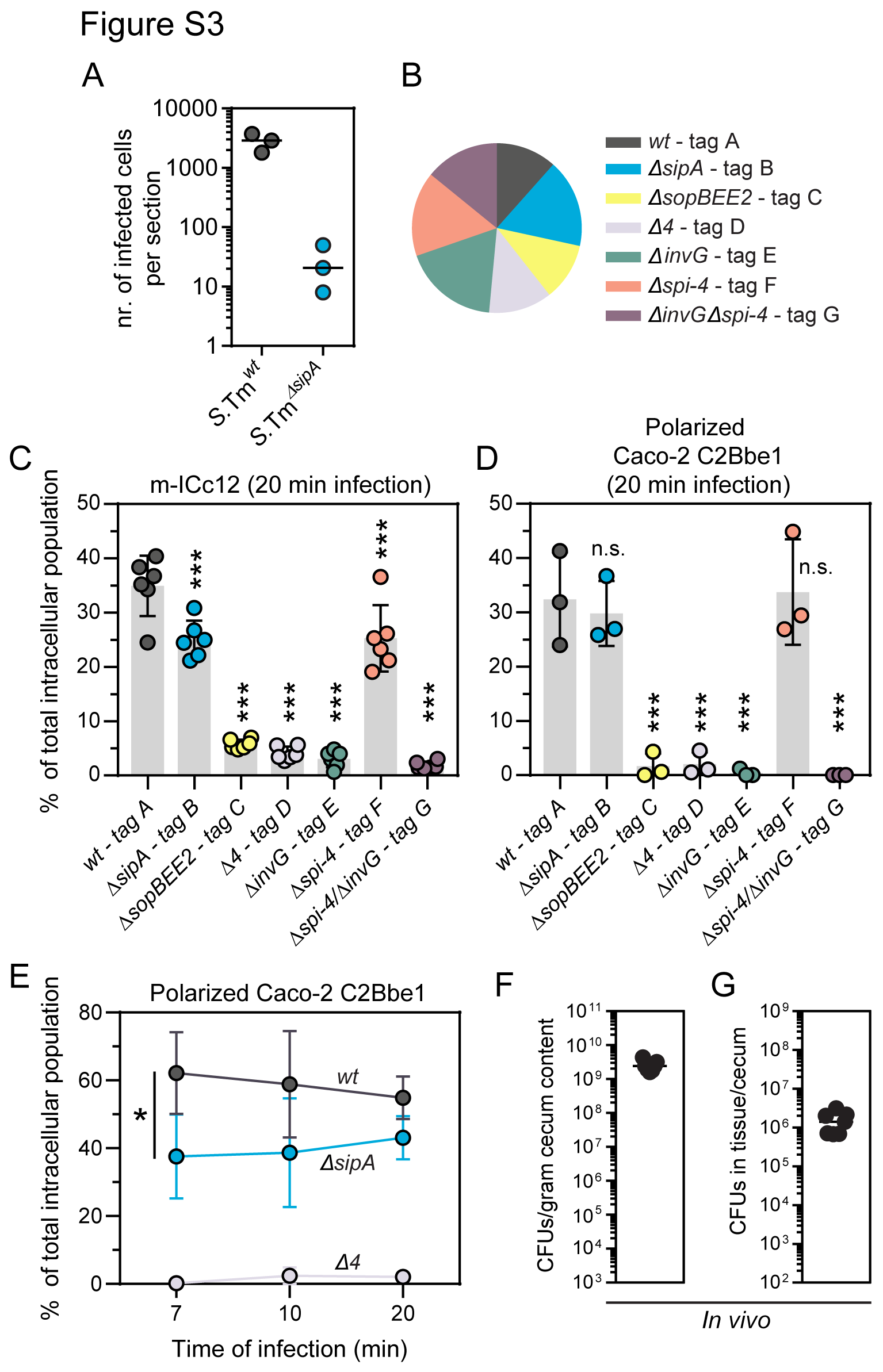

Supplement: S3 Fig — (A) Dependence on SipA for S.Tm absorptive epithelial cell invasion in vivo. Inflammasome-deficient (Nlrc4-/-) mice were orally infected with the indicated S.Tm/pssaG-GFP strains for 18h. Graph shows quantification of the number of intraepithelial S.Tm foci per 20μm section (mice also analyzed for total intraepithelial S.Tm loads in Fig 2C). Each data point corresponds to one animal. Line at median. (B-G) Barcoded consortium infections of epithelial cell lines and in vivo in mice. (B) Relative abundance of the individual strains in the barcoded consortium inoculum. The pie chart depicts the average from seven replicate experiments, where the relative abundance of each strain was assessed by quantitative PCR after enrichment culture. Note that none of the strains in the consortium is significantly over/underrepresented in the inoculum. (C-D) Barcoded consortium infections of (C) m-ICc12 cells on plastic, and (D) polarized Caco-2 C2Bbe1 cells grown atop Transwell inserts. The cells were infected for 20min at a total MOI of 2, using the same seven strain barcoded consortium as in Fig 2F and S3B Fig. Bars correspond to mean +/- SD of six (C) or three (D) replicate infections (circle symbols). (E) Barcoded consortium infection of polarized Caco-2 C2Bbe1 cells grown atop Transwell inserts with a less complex consortium. The cells were infected for 7, 10 or 20min at a total MOI of 2, using a barcoded consortium containing six tagged strains; two S.Tmwt (tag C and tag D), two S.TmΔsipA (tag B and tag F), and two S.TmΔ4 strains (tag E and tag G) (see S1 Table). The relative abundance for S.Tmwt, S.TmΔsipA, and S.TmΔ4 was calculated based on the summed abundance of the two internal technical replicates for each strain. Data points correspond to mean +/- SD of three replicate infections with separately prepared consortia. In C-E, One-way ANOVA with Dunnett´s test (n.s., not significant; *p<0.05, ***p<0.001). (F-G) Total S.Tm CFU counts in cecum content (F) and washed cecal tis [file ppat.1008503.s003.tif]

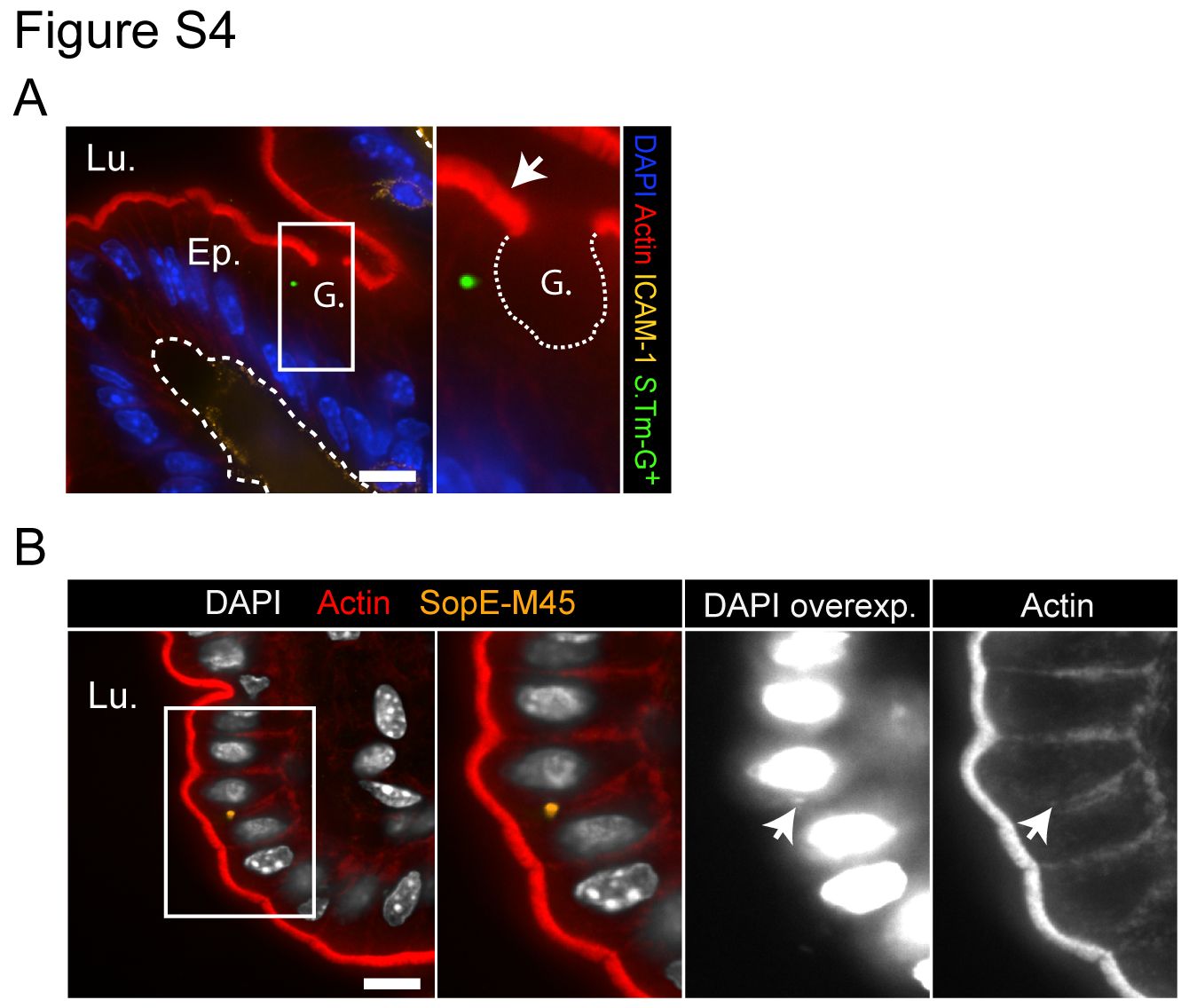

Supplement: S4 Fig — (A-B) Additional fluorescence micrographs of early S.Tm invasion into gut absorptive epithelial cells in vivo in mice. (A) Additional representative micrographs of the cecal epithelium in wild-type mice orally infected with S.Tmwt/pssaG-GFP for 6h, as in Fig 3F. Blow-up shows magnification of boxed region. Lu.–Lumen; Ep.–Epithelium; G.–Goblet cell. White arrow indicates the apical actin brush border of an infected absorptive epithelial cell. Scale bar: 10μm. (B) Additional representative micrographs of a SopE-M45 positive focus in the cecal epithelium in Rag1-/- mice orally infected with S.Tmwt/psopE-M45 for 8h, as in Fig 3H. Blow-up shows magnification of boxed region. Lu.—Lumen. White arrow indicates an M45-positive bacterial focus. Scale bar: 10μm. (TIF) [file ppat.1008503.s004.tif]

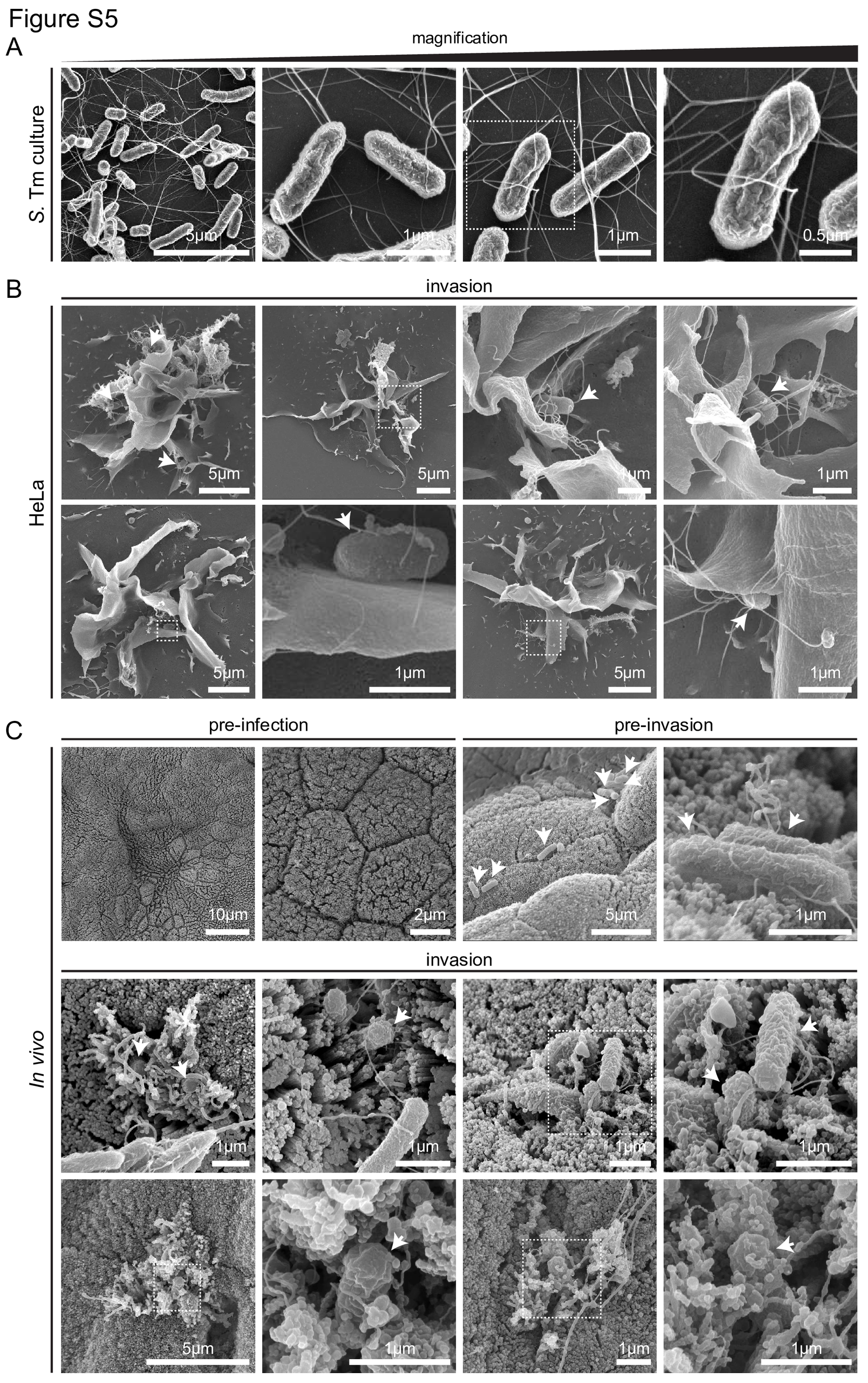

Supplement: S5 Fig — (A-C) Additional SEM micrographs of S.Tm invasion into epithelial cell lines and the absorptive gut epithelium in vivo in mice. (A) SEM micrographs of the S.Tmwt inoculum used in Fig 3J–3L. (B) Additional SEM micrographs of HeLa cells infected with S.Tmwt for 6-10min at MOI 400, as in Fig 3J. (C) Additional SEM micrographs of the cecal epithelium in mice, either uninfected, or upon infection with S.Tmwt, as in Fig 3L. Scale bars indicated separately for each panel. Arrow heads point to S.Tm. For each micrograph containing a dashed white box, the panel directly to the right of it represents the same area at higher magnification. (TIF) [file ppat.1008503.s005.tif]

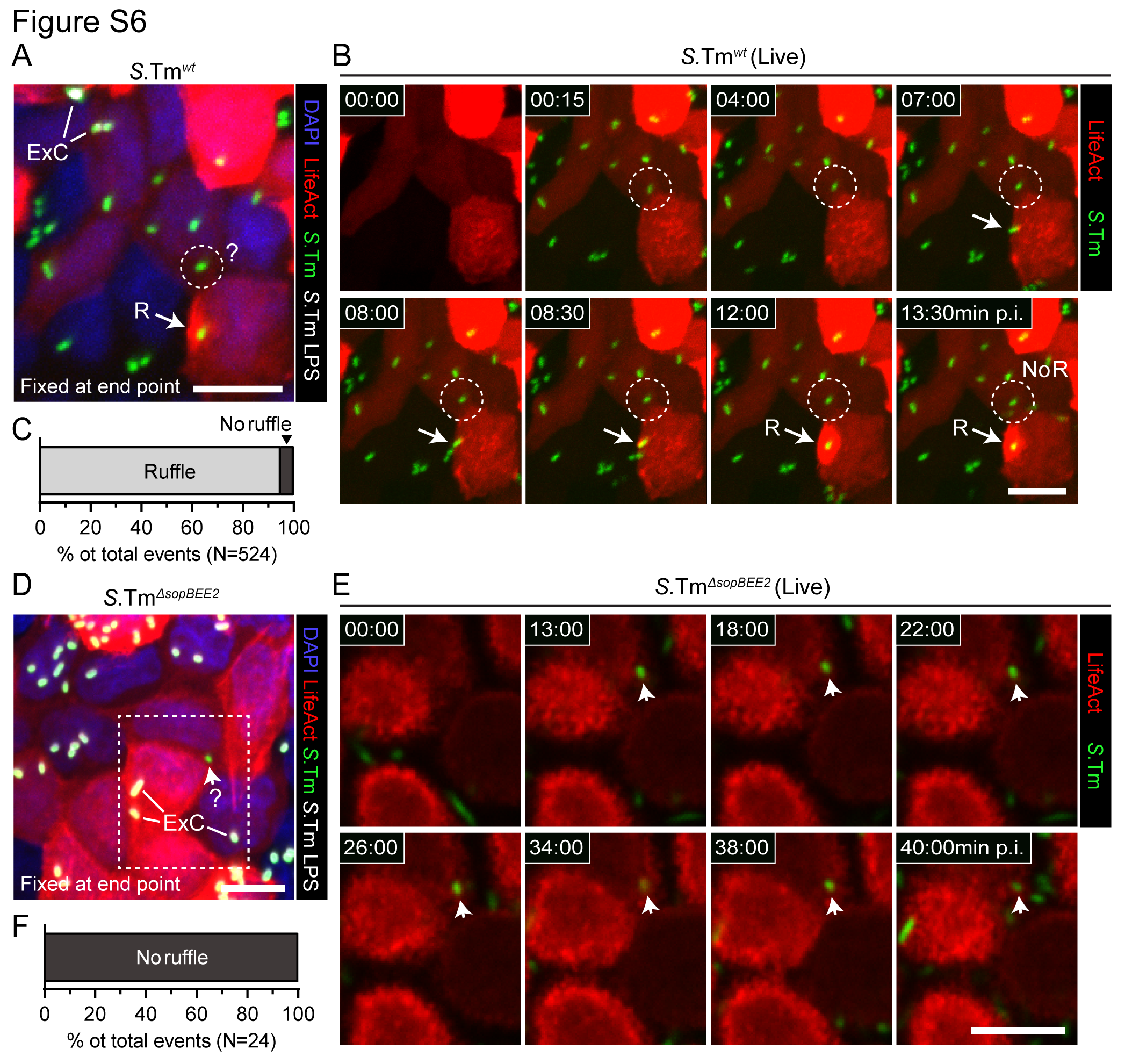

Supplement: S6 Fig — (A-C) S.Tmwt invades a polarized epithelial cell line predominantly, but not exclusively, through induction of visible actin ruffles. Polarized LifeAct-expressing MDCK cells (red) were infected with S.Tmwt/pmCherry (green) at MOI 50. (A) Micrograph of cells fixed at the end point (14min p.i.). ExC—extracellular bacterium; R—ruffle;? —ambiguous entry event followed in the live series. (B) Live imaging series preceding A. R—ruffle; No R—no ruffle (encircled). (C) Quantification of the presence/absence of visible actin ruffles at S.Tmwt entry sites. Ntot = 524 invasion events analyzed. (D-F) S.TmΔsopBEE2 consistently invades a polarized epithelial cell line without triggering visible actin ruffles. Polarized LifeAct-expressing MDCK cells (red) were infected with S.TmΔsopBEE2/pmCherry (green) at MOI 500. (D) Micrograph of cells fixed at the end point (40min p.i.). ExC–extracellular bacterium;? —ambiguous entry event followed in the live series. (E) Live imaging series of boxed region preceding D. Arrow head indicates a ruffle-less entry event. (F) Quantification of the presence/absence of visible actin ruffles at S.TmΔsopBEE2 entry sites. Ntot = 24 invasion events analyzed. Scale bars: 10μm. (TIF) [file ppat.1008503.s006.tif]
