# Supplementary material for: Salmonella Typhimurium discreet-invasion of the murine gut absorptive epithelium
Source: PLoS Pathog. 2020 May 4;16(5):e1008503. doi: 10.1371/journal.ppat.1008503 (PMC7224572; doi:10.1371/journal.ppat.1008503)
Supplement: S1 Table — (DOCX) [file ppat.1008503.s015.docx]

Table S1. Bacterial strains and plasmids used in this study.

| *Salmonella enterica* Typhimurium SL1344 strains | | | |
| --- | --- | --- | --- |
| Name in this study | **Alternative name** | **Genotype** | **Reference** |
| *S*.Tm*^wt^* | SB300 | *SL1344 wild-type* | *Hoiseth et al. 1981* |
| *S*.Tm*^ΔinvG^* | SB161 | *ΔinvG* | *Kaniga et al. 1994* |
| *S*.Tm*^Δspi-4^* | SB300Δspi-4 | *Δspi-4* | *Gerlach et al. 2008* |
| *S*.Tm*^ΔsipA^* | M714 | *ΔsipA* | *Hapfelmeier et al. 2004* |
| *S*.Tm*^ΔsopBEE2^* | M516 | *ΔsopB, sopE::aphT, sopE2::tet* | *Mirold et al. 2001* |
| *S.Tm^Δ4^* | M566 | *ΔsopB, ΔsopE, ΔsopE2, ΔsipA* | *Ehrbar et al. 2003* |
| *S*.Tm*^"wt"^* TTSS-2 neg. | M556 | *sseD::aphT* | *Hapfelmeier et al. 2004* |
| *S*.Tm*^ΔsipA^* TTSS-2 neg. | M715 | *ΔsipA, sseD::aphT* | *Hapfelmeier et al. 2004* |
| *S*.Tm*^ΔsopBEE2^* TTSS-2 neg. | M716 | *ΔsopB, sopE::aphT, sopE2::tet, sseD::aphT* | *Hapfelmeier et al. 2004* |
| *S*.Tm*^wt^*-tag A | SB300-tag A | *SL1344 wild-type,*  *malXY::tagA* | *Di Martino et al. 2019* |
| *S*.Tm*^ΔsipA^*-tag B | M714-tag B | *ΔsipA, malXY::tagB* | This study* |
| *S*.Tm*^ΔsopBEE2^*-tag C | M516-tag C | *ΔsopB, sopE::aphT, sopE2::tet, malXY::tagC* | *Di Martino et al. 2019* |
| *S*.Tm*^Δ4^*-tag D | M566-tag D | *ΔsopB, ΔsopE, ΔsopE2, ΔsipA, malXY::tagD* | *Di Martino et al. 2019* |
| *S*.Tm*^ΔinvG^*-tag E | SB161-tag E | *ΔinvG, malXY::tagE* | *Di Martino et al. 2019* |
| *S*.Tm*^Δspi-4^*-tag F | SB300Δspi-4-tag F | *Δspi-4,*  *malXY::tagF* | This study* |
| *S*.Tm*^ΔinvGΔspi-4^*-tag G | SB300ΔinvGΔspi-4-tag G | *ΔinvG, Δspi-4,*  *malXY::tagG* | This study* |
| *S*.Tm*^wt^*-tag C | SB300-tag C | *SL1344 wild-type,*  *malXY::tagC* | *Di Martino et al. 2019* |
| *S*.Tm*^wt^*-tag D | SB300-tag D | *SL1344 wild-type,*  *malXY::tagD* | *Di Martino et al. 2019* |
| *S*.Tm*^ΔsipA^*-tag F | M714-tag F | *ΔsipA, malXY::tagF* | *Di Martino et al. 2019* |
| *S*.Tm*^Δ4^*-tag E | M566-tag E | *ΔsopB, ΔsopE, ΔsopE2, ΔsipA, malXY::tagE* | This study* |
| *S*.Tm*^Δ4^*-tag G | M566-tag G | *ΔsopB, ΔsopE, ΔsopE2, ΔsipA, malXY::tagG* | This study* |
| Plasmids | | | |
| Name in this study | **Alternative name** | **Plasmid genotype** | **Reference** |
| p*GFP* | pM965 | *rpsM-gfp* | Stecher *et al*. 2004 |
| p*mCherry* | pFPV25-mCherry | *rpsM-mCherry* | *Drektah et al. 2008* |
| p*ssaG-GFP* | pM975 | *ssaG-gfp* | *Hapfelmeier et al. 2005* |
| p*ssaG-mCherry* | pZ400 | *ssaG-mCherry* | *Sellin et al. 2014* |
| p*sipA-M45* | pZ923 | *sipA-M45* | This study** |
| p*sopE-M45* | pM438 | *sopE-M45* | *Ehrbar et al. 2003* |

*Construction of barcoded *S*.Tm strains: The ~40 nucleotide genetic barcodes placed within the *S*.Tm malXY locus have been described and validated before (Grant et al. 2008). Barcode sequences are given in Table S2. The barcodes were transferred from reservoir *S*.Tm strains into the relevant wild-type or mutant strain by p22 transduction, followed by selection on LB agar containing 12.5μg/ml chloramphenicol.

**Construction of p*sipA-M45*: A *sipA-M45* fragment was cut out of plasmid pM1301, using restriction enzymes EheI and SalI. The fragment was gel purified and ligated into a Eco321/SalI-digested pACYC184 vector, leaving the chloramphenicol resistance cassette intact. The ligation mix was transformed into *E. coli* CC118, and colonies screened by PCR. Positive colonies were enriched, the plasmid purified and confirmed by nucleotide sequencing.
