# Supplementary material for: Salmonella Typhimurium discreet-invasion of the murine gut absorptive epithelium
Source: PLoS Pathog. 2020 May 4;16(5):e1008503. doi: 10.1371/journal.ppat.1008503 (PMC7224572; doi:10.1371/journal.ppat.1008503)
Supplement: S2 Table — (DOCX) [file ppat.1008503.s016.docx]

Table S2. Genetic barcodes and PCR primers for detection. Barcodes and primer sequences were adopted from (*Grant et al.* *2008*).

| Barcode name | Barcode sequence | Primer 1 (5’- 3’)  (common) | Primer 2 (5’- 3’) |
| --- | --- | --- | --- |
| Tag A | cgcgcgataggtgtggagtggtgtcgttgggggggttgtt | ggctgtccgcaatgggtc | acgacaccactccacaccta |
| Tag B | ggggagttgttggtattgcgggtggtagctggtgggagct | ggctgtccgcaatgggtc | acccgcaataccaacaactc |
| Tag C | ggcgagggcgcgagtgagatcgagtgtgtgggattgatat | ggctgtccgcaatgggtc | atcccacacactcgatctca |
| Tag D | agctggagctcgagagtgagtgaggggtgtctttagctat | ggctgtccgcaatgggtc | gctaaagacacccctcactca |
| Tag E | ggcgtgggagtgagggggtgggctggtgagcgttatagtt | ggctgtccgcaatgggtc | tcaccagcccaccccctca |
| Tag F | aggggtggggttatggggctggatagtgcgcgtgctagct | ggctgtccgcaatgggtc | gcactatccagccccataac |
| Tag G | agagagagctggagagtgatcggtggttgtggtgggagtg | ggctgtccgcaatgggtc | acaaccaccgatcactctcc |
